# Cryo-EM structure of the vaccinia virus entry fusion complex reveals a multicomponent fusion machinery

**DOI:** 10.1126/sciadv.aec0254

**Published:** 2026-01-14

**Authors:** Chang Sheng-Huei Lin, Ching-An Li, Chun-Hsiung Wang, Chi-Fei Kao, Hsiao-Jung Chiu, Min-Chi Yeh, Hua-De Gao, Meng-Chiao Ho, Hsien-Ming Lee, Wen Chang

**Affiliations:** ^1^ Institute of Molecular Biology, Academia Sinica, Taipei, Taiwan, R.O.C.; ^2^ Institute of Biological Chemistry, Academia Sinica, Taipei, Taiwan, R.O.C.; ^3^ Molecular and Cell Biology, Taiwan International Graduate Program, Academia Sinica and Graduate Institute of Life Sciences, National Defense Medical Center, Taipei, Taiwan, R.O.C.; ^4^ Institute of Chemistry, Academia Sinica, Taipei, Taiwan, R.O.C.

## Abstract

Membrane fusion is essential for viral entry. Unlike class I-III fusion proteins, vaccinia virus (VACV) uses a multicomponent entry fusion complex (EFC). Using cryo–electron microscopy, we determined the full-length structure of the VACV EFC at near-atomic resolution, revealing a 15-protein asymmetric assembly organized into three layers. The central A16/G9/J5 heterotrimer forms the fusion core, stabilized by conserved PXXCW and Delta motifs, and anchors two A28/H2 adaptor dimers linked to peripheral G3/L5/A21/O3 scaffolds. Structural and evolutionary analyses identify a conserved N-terminal domain in A16 containing a myristoyl-binding pocket and a phenylalanine-rich region that stabilizes the trimer and may regulate lipid engagement. An additional component, F9, binds peripherally to J5, A21, and H2 through Delta-like motifs, reinforcing the prefusion architecture. Together, these results define the VACV EFC as a unique multiprotein fusion machinery and provide a structural framework for understanding the mechanism of poxvirus entry and membrane fusion.

## Teaser

Structure of vaccinia entry fusion complex reveals a unique multiprotein architecture for poxvirus entry.

## INTRODUCTION

Vaccinia virus (VACV), an enveloped DNA virus of the *Poxviridae* family, is best known for its pivotal role in eradicating smallpox and providing cross-protection against Mpox (*1*). Its large ~190-kb genome encodes more than 200 proteins (*2*). Unlike other DNA viruses, VACV replicates entirely in the cytoplasm, producing two infectious forms of viral particles, the mature virus (MV) and the enveloped virus (EV) (*3*). MV remains intracellular until cell lysis, while EV acquires an additional membrane from the trans-Golgi network and is released extracellularly. Both forms of virus particles require an identical fusion machinery to fuse viral membrane with the host membrane during cell entry. Furthermore, to ensure that membrane fusion occurs at the correct time and location, VACV has evolved two distinct sets of viral fusion inhibitors (*4*, *5*). The A26 protein, associated with MV, suppresses premature membrane fusion and regulates low pH-dependent fusion during endocytosis (*6*, *7*). Deletion of A26 enables MVs to fuse directly with the plasma membrane in a pH-independent manner, indicating that A26 acts as an acid-sensitive fusion suppressor that links environmental pH sensing to activation of VACV EFC. On the other hand, the A56/K2 complex, expressed on the surface of infected cells, prevents superinfection by blocking EV-mediated fusion with already infected cells (*4*, *8*).

From studies of all EV entry and membrane fusion processes, a general scheme for viral membrane fusion has emerged. The membrane fusion process generally involves conformational changes in the viral fusion protein, transitioning from a prefusion state to a postfusion state (*9*). In the prefusion state, the fusion peptide or loop is embedded within the protein. When the pH drops from neutral to acidic, the fusion protein undergoes sequential conformational changes that enable the fusogenic region to insert into the host membrane, initiate hemifusion, and ultimately complete membrane fusion between the host and viral membranes. The fusion protein adopts the postfusion conformation once membrane fusion is achieved. Whereas classes I (*10*, *11*), II (*12*–*14*), and III (*15*, *16*) viral fusion mechanisms have been elucidated largely through crystal structures of individual fusogens, the fusion machinery of VACV remains to be characterized.

The membrane fusion of VACV is facilitated by a multiprotein entry fusion complex (EFC) (*17*, *18*). VACV EFC comprising 11 conserved, nonglycosylated transmembrane (TM) proteins: A16 (OPG143), A21 (OPG147), A28 (OPG155), F9 (OPG053) G3 (OPG086), G9 (OPG094), H2 (OPG107), J5 (OPG104), L1 (OPG095), L5 (OPG099), and O3 (OPG076) (*17*, *18*). Previous coimmunoprecipitation studies using conditional-lethal mutants suggest that nine EFC proteins, A16 (*19*), A21 (*20*), A28 (*21*), G3 (*22*), G9 (*23*), H2 (*24*), J5 (*25*), L5 (*26*), and O3 (*27*), form a stable major complex whereas the remaining L1 (*28*) and F9 (*29*) function as peripheral components. Distinct subcomplexes, including A16/G9 (*30*), A28/H2 (*17*, *31*), and G3/L5 (*32*) have been identified in the infected cells, and structures of all individual EFC proteins have been determined experimentally by x-ray crystallography [A16/G9 (*33*), G3/L5 (*34*), H2 (*35*), F9 (*36*), L1 (*37*), and A21 (*38*)], nuclear magnetic resonance (NMR) spectroscopy [A28 (*39*) and J5 (*40*)], or predicted by AlphaFold modeling (*41*). Recently, the cryo–electron microscopy (cryo-EM) structure of A16/G9 bound to the fusion suppressor A56/K2 was reported, providing a partial view of the regulatory interface (*42*). However, the complete architecture, stoichiometry, and assembly principles of the EFC remain unresolved (*18*, *41*, *43*). In this study, we present the full-length cryo-EM structure of the VACV EFC, defining its structural organization and revealing the molecular interactions among its components that underlie its unique role in membrane fusion.

## RESULTS

### Cryo-EM structure of the pre-fusion VACV EFC reveals a 15-protein assembly

**Fig. 1.**
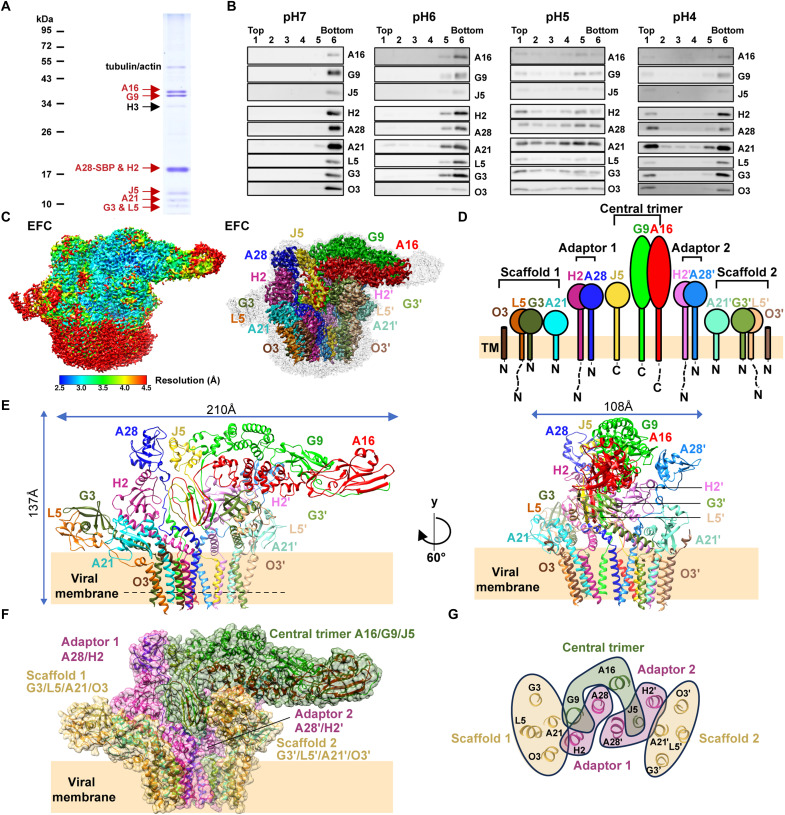
Cryo-EM structure of the pre-fusion VACV EFC. (**A**) SDS-PAGE analysis of purified VACV EFC eluted from the streptavidin affinity column. Major EFC components are indicated; contaminating host proteins (tubulin/actin) and the viral membrane protein H3 are marked. Molecular weight markers are shown at left. (**B**) In vitro liposome coflotation assay of purified EFC after neutral or acidic pH treatment. Purified EFC and liposomes were mixed at different pH levels from 7 to 4 for 2 hours, followed by OptiPrep gradient centrifugation and collected into six fractions (top to bottom). The distribution of each EFC component was analyzed by immunoblotting. (**C**) Cryo-EM density map of the EFC. Left: map colored by local resolution and contoured at 2.5σ. Right: map contoured at 6σ and colored by subunit identity, A16 (red), G9 (green), J5 (yellow), A28 (blue), A28′ (light blue), H2 (magenta), H2′ (light pink), G3 (forest green), G3′ (light green), L5 (orange), L5′ (light orange), A21 (cyan), A21′ (light cyan), O3 (brown), and O3′ (light brown) are highlighted (right). (**D**) Schematic diagram showing the topology of the 15-protein EFC and its tripartite architecture: the central trimer, two adaptor pairs, and two scaffold assemblies. Single-pass transmembrane (TM) helices are indicated by vertical bars. Colors correspond to those in (C). (**E**) Atomic model of the 15-protein EFC viewed from two orientations. The model was reconstructed by fitting AlphaFold predicted subunits and manually rebuilding them into the cryo-EM density map, as shown in (C). Approximate dimensions are indicated. (**F**) Surface representation highlighting the tripartite organization: the central trimer (green), adaptor 1/2 (magenta), and scaffold 1/2 (beige). (**G**) Cross-sectional cartoon of TM domains along the dashed line in (E), showing the spatial arrangement of the central trimer, adaptors, and scaffolds. Color coding follows (F).

The VACV EFC comprises 11 viral membrane proteins, A28, H2, A16, G9, J5, G3, L5, A21, O3, F9, and L1, that collectively mediate membrane fusion during viral entry (fig. S1). To isolate the native EFC, HeLa cells were infected with a recombinant VACV expressing A28 tagged at the C terminus with a streptavidin-binding peptide (vA28-SBP). Streptavidin affinity purification yielded a single elution peak (Fig. 1A). Protein bands visible on SDS–polyacrylamide gel electrophoresis (PAGE) were excised and analyzed by mass spectrometry, confirming the presence of eight components, A16, G9, A28, H2, J5, A21, G3, and L5 in the purified EFC sample. The final component, O3, is a small (~4.2 kDa) and highly hydrophobic protein that likely escaped detection by these methods (*44*–*46*). Minor contaminants, including tubulin, actin, and a VACV H3 protein, were also detected, but their levels varied among experiments.

To assess whether the purified EFC adopts a prefusion conformation, we performed a protein-liposome coflotation assay across a pH gradient, a method previously used to evaluate membrane insertion of viral fusion proteins (*47*, *48*). Synthesized liposomes (fig. S2) were incubated with the EFC and subjected to different pH conditions ranging from 7 to 4. After differential centrifugation (*48*), fractions were collected from top to bottom and analyzed by immunoblotting to determine the distribution of EFC components. As shown in Fig. 1B, at neutral pH (7.0), all EFC proteins localized to the bottom fraction (fraction 6), consistent with a non–liposome-associated prefusion state. At pH 6.0, the EFC components began migrating upward in the gradient, indicating the partial liposome engagement. At pH 5.0, all EFC proteins cofloated with liposomes and spread across the upper fractions. At pH 4.0, the EFC proteins concentrated in the top layer, although some residual proteins remained at the bottom, likely reflecting partial aggregation under strongly acidic conditions. These findings demonstrate a pH-dependent membrane association of the EFC, supporting the notion that the purified EFC is in a prefusion state and can undergo an acid-triggered transition that promotes membrane engagement, consistent with the low pH–mediated activation mechanism of viral fusion proteins during cell entry.

Single-particle cryo-EM of purified VACV EFC revealed two distinct structure classes: the EFC alone (Fig. 1C, figs. S3 to S5, table S1, and movie S1) and an F9-containing assembly (EFC + F9), the latter will be discussed in a later paragraph. Three-dimensional reconstruction of the EFC yielded a density map at an overall resolution of 2.98 Å (Fig. 1C, contoured at 2.5σ in the left panel and at 6σ in the right). The map displayed well-resolved densities for 15 proteins, including one copy of A16, G9, and J5, and two copies of A28, H2, G3, L5, A21, and O3 (Fig. 1, D and E, and fig. S6). The reconstructed VACV EFC measures approximately 210 Å by 137 Å by 108 Å (Fig. 1E) and adopts a bouquet-like morphology, with the TM helices forming a bundled “stem” and the ectodomains extending outward to create a “blooming” cap.

The EFC is organized into three hierarchical layers: (i) central trimer, (ii) adaptors, and (iii) peripheral scaffolds (Fig. 1F). The known A16/G9 subcomplex forms a central heterotrimer with J5, which is approximately one-third the size of A16/G9. This skewed central trimer is flanked by two A28/H2 heterodimers, forming the middle layer of the adaptor complex. These adaptors bridge the central heterotrimer to two peripheral heterotetramers, each composed of G3/L5, A21, and O3, which constitute the outermost scaffold layer of the EFC (Fig. 1, E and F). No symmetry pairs were identified within the EFC. The A16/G9/J5 heterotrimer is present as a single asymmetric unit. The two copies of the A28/H2 adaptors adopt distinct conformations and cannot be superimposed, as detailed in subsequent sections. Likewise, structural overlays of the two G3/L5 copies, as well as the complete G3/L5/A21/O3 scaffold subcomplexes, were unsuccessful due to pronounced conformational differences. Last, the TM helices are organized as intertwined interaction networks embedded within the viral membrane (Fig. 1G). At the two lateral edges, the TM helices from the two scaffolds form four-helix bundles. In each bundle, A21 is positioned at the inner edge, acting as a linker between the scaffold, the adaptors, and the central trimer. Besides, the TM helix of G9 sits between the A28/H2 adaptor 1, while the TM helix of J5 is inserted between the A28′/H2′ adaptor 2. This intercalated arrangement effectively clamps the adaptors’ TM helices, reinforcing their association within the central trimer TM helices. Together, our cryo-EM structure reveals that VACV pre-fusion EFC adopts a three-layer asymmetric architecture consisting of 15 proteins and conforms to C1 symmetry. The TM helices form a tightly packed membrane-embedded helical bundle anchoring the EFC in the viral membrane.

### Asymmetric hinge flexibility governs intra-subcomplex interactions within adaptor and scaffold modules of the EFC

**Fig. 2.**
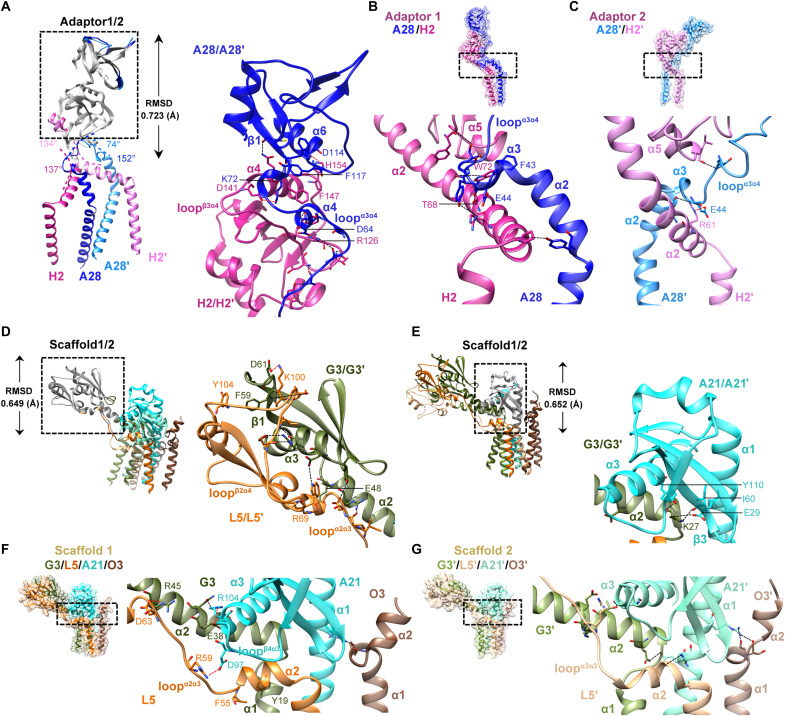
Asymmetric organization and hinge flexibility of adaptor and scaffold subcomplexes within the EFC. [(A), (D), and (E)] Superimposed structures of adaptor and scaffold pairs reveal conserved cores and asymmetric features. (**A**) Alignment of adaptor 1 (A28/H2) and adaptor 2 (A28′/H2′), with aligned regions shown in gray (RMSD = 0.72 Å). The boxed close-up highlights the secondary structures and conserved contact residues at the A28-H2 and A28′-H2′ interfaces. (**B** and **C**) Structures of adaptor 1 and adaptor 2, respectively, with boxed close-ups showing distinct hinge-contact residues. (**D**) Comparison of scaffold 1 (G3/L5) and scaffold 2 (G3′/L5′) (RMSD = 0.65 Å), showing shared contacts in gray. The inset highlights the secondary structures and contact residues mediating at the G3-L5 and G3′-L5′ interfaces. (**E**) Alignment of A21 and A21′ within scaffold 1 and scaffold 2 (RMSD = 0.65 Å), with common intersubunit contacts shown in gray. The inset highlights the secondary structures and contact residues mediating at the A21-G3/L5 and A21′-G3′L5′ interfaces. The unaligned regions in each panel are colored as in Fig. 1C. [(B), (C), (F), and (G)] Structural divergence between the two adaptor and scaffold pairs. (**F** and **G**) Structures of scaffold 1 (G3/L5/A21/O3) and scaffold 2 (G3′/L5′/A21′/O3′), with insets highlighting secondary structures and differences in hydrogen bonding and ionic interactions at their hinge interfaces. Residues are shown as sticks and labeled. Hydrogen bonds and salt bridges are represented by dashed black and red lines, respectively.

To understand how identical subunits assemble into an asymmetric architecture, we compared equivalent adaptors and scaffolds by structural alignment. Superposition of the two adaptor heterodimers (A28/H2 and A28′/H2′) yielded a root mean square deviation (RMSD) of 0.723 Å within the ectodomains (Fig. 2A and fig. S7A). Comparison with previously solved NMR and x-ray structures (*35*, *39*) confirmed that A28 and H2 retain conserved ectodomain folds (fig. S8), indicating a stable, preassembled heterodimeric module. The A28/H2 interface [~1820 Å^2^, obtained from proteins, interfaces, structures and assemblies (PISA) analysis] involves α4, α6, β1, and loop^α3α4^ of A28/A28′ and α4 plus loop^β3α4^ of H2/H2′, stabilized by three salt bridges (A28/A28′^D64^-H2/H2′^R126^, A28/A28′^K72^-H2/H2′^D141^, and A28/A28′^D114^-H2/H2′^H154^), a π-π interaction (A28/A28′^F117^-H2/H2′^F147^), and multiple hydrogen bonds (Fig. 2A, dashed lines). Despite these conserved ectodomains, the hinge regions, located in α2, α3, and loop ^α3α4^ of A28/A28′, and α2 and α5 of H2/H2′, adopt distinct conformations with unique interactions (Fig. 2, B and C). In adaptor 1, A28^E44^ forms a hydrogen bond with H2^T68^ (Fig. 2B), while in adaptor 2, A28′^E44^ forms a salt bridge with H2′^R61^ (Fig. 2C). A π-π stacking between A28^F43^ and H2^W72^ occurs only in adaptor 1 (Fig. 2B). These hinge differences explain the asymmetric engagement of A28/H2 and A28′/H2′ with the central A16/G9/J5 trimer.

The two scaffolds (G3/L5/A21/O3 and G3′/L5′/A21′/O3′) display similar principles. Although scaffolds 1 and 2 maintain conserved ectodomain folds (Fig. 2, D and E), their hinge regions differ markedly (Fig. 2, F and G). RMSD values for G3/L5 and G3′/L5′ dimers, A21/A21′, and O3/O3′ were 0.649, 0.659, and 0.456 Å, respectively (fig. S7A), consistent with previous crystal structures (fig. S8) (*34*, *38*). The G3/L5 and G3′/L5′ interfaces (~1760 Å^2^) involve α2, α3, and β1 of G3/G3′, and loops^α2α3 & β2α4^ of L5/L5′, stabilized by two salt bridges (G3/G3′^D61^-L5/L5′^K100^ and G3/G3′^E48^-L5/L5′^R69^), a π-π stack (G3/G3′^F59^-L5/L5′^Y104^), and hydrogen bonds (Fig. 2D), consistent with a self-contained scaffold module (*32*). Similarly, the α1, α3, and β3 of A21/A21′ associate with α2 of G3/G3′ in G3/L5 and G3′/L5′, via conserved contacts between G3/G3′^K27^ and A21/A21′^E29^, A21/A21′^I60^, and A21/A21′^Y110^, respectively (Fig. 2E), whereas distinct interactions among α1and α2 of G3/G3′, α2 of L5/L5′and α1 and α3 of A21/A21′, define hinge-specific conformations (Fig. 2, F and G). The scaffold 1 hinge features three salt bridges (G3^R45^-L5^D63^, G3^E38^-A21^R104^, and L5^R59^-A21^D97^), a π-π stack (G3^Y19^-L5^F55^), and hydrogen bonds (Fig. 2F), whereas scaffold 2 hinge adopts an alternative hydrogen-bonding pattern (Fig. 2G). The last component O3/O3′ of scaffold 1/2 also contributes to unique hinge interactions through hydrogen bonds with A21/A21′ (Fig. 2, F and G), respectively. Together, these findings demonstrate that hinge flexibility, rather than ectodomain architecture, drives the asymmetric assembly of otherwise identical adaptor and scaffold modules within the EFC, consistent with mutational evidence implicating hinge residues in EFC function (fig. S7B and table S2) (*35*, *39*, *40*, *49*).

### Intersubcomplex interfaces integrate the central trimer, adaptor, and scaffold layers of the EFC

**Fig. 3.**
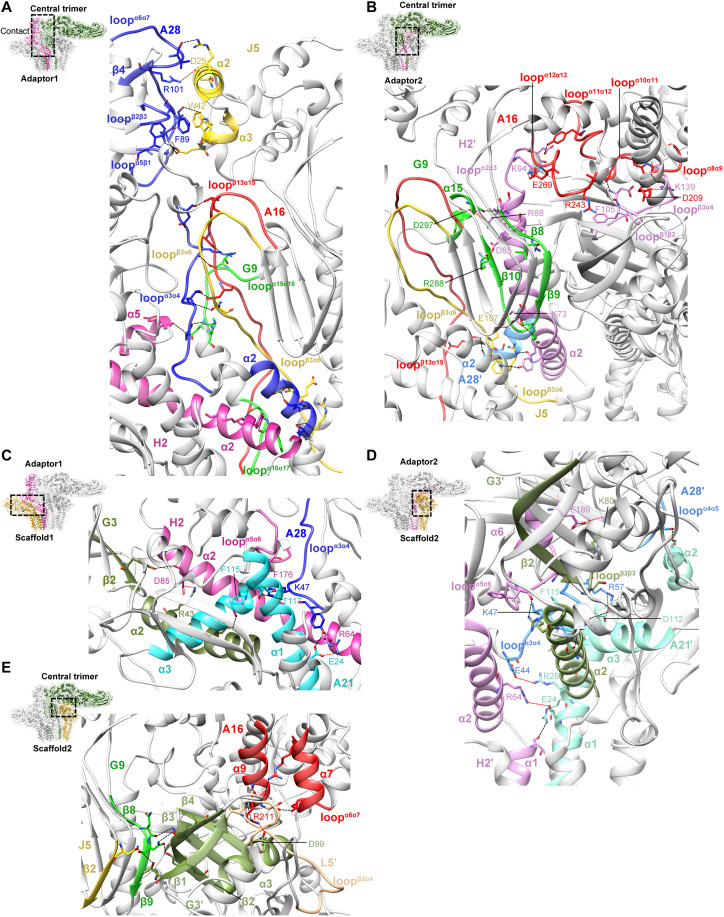
Intersubcomplex interfaces connecting adaptor, scaffold, and trimer layers within the EFC ectodomain. [(A) to (E)] Structural interfaces linking the central trimer with the surrounding adaptor and scaffold subcomplexes. (**A**) Interface between the central trimer and adaptor 1. (**B**) Interface between the central trimer and adaptor 2. (**C**) Interface between adaptor 1 and scaffold 1. (**D**) Interface between adaptor 2 and scaffold 2. (**E**) Interface between the central trimer and scaffold 2. In each panel, the contact interface between two subcomplexes is boxed, with a close-up view showing secondary structures and interacting residues. Residues involved in intersubcomplex contacts are shown as sticks and labeled. Hydrogen bonds and salt bridges are represented by dashed black and red lines, respectively. Proteins are color-coded as in Fig. 1C.

To define the interfaces mediating EFC assembly, we analyzed the contact surfaces among the ectodomains of the central trimer, adaptors, and scaffolds using PISA (*50*) and RING (*51*). The adaptor 1–central trimer interface spans 2481 Å^2^ (Fig. 3A and fig. S9A), with contacts extending from the ectodomains of A28 and J5 through midregion loops to the membrane-proximal hinge regions (Fig. 3A). The upper contact region involves A28 loops^α5β1& β2β3 & α6α7 & β4^ interacting with J5^α2 & α3^, stabilized by a salt bridge (A28^R101^-J5^D25^), a π-π stack (A28^F89^-J5^W42^), and hydrogen bonds (Fig. 3A, dashed lines). The middle contact region, formed by A28 loop^α3α4^, A16 loop^β13α15^, J5 loop^β3α6^, G9 loop^α15α16^, and H2 ^α5^, contains an extensive hydrogen bond network. The lower hinge interface, involving A28^α2^, H2^α2^, G9 loop^α16α17^, and J5 loop^β3α6^, is also dominated by hydrogen bonding (Fig. 3A).

The adaptor 2–central trimer interface covers 1579 Å^2^ (Fig. 3B and fig. S9B) and is primarily of electrostatic interactions. The upper contact, between H2′ loops^α2α3 & β1β2 & β3α4^ and A16 loops^α8α9 & α10α11 & α11α12 & α12α13^, includes two salt bridges (H2′^K94^-A16^E269^ and H2′^K139^-A16^D209^), a π-cation interaction (H2′^F105^-A16^R243^), and multiple hydrogen bonds (Fig. 3B, dashed lines). The middle contact, between H2′^α2^ and G9^β8-β10 & α15^, contributes two additional salt bridges (H2′^D85^-G9^R288^ and H2′^R88^-G9^D297^) and several hydrogen bonds. A hinge-proximal contact between J5 loop^β3α6^and H2′^α2^ adds another salt bridge (H2′^K73^-J5^E107^) and additional hydrogen bonds (Fig. 3B).

The adaptor 1–scaffold 1 interface (Fig. 3C and fig. S9C) has an area of 839 Å^2^, involving G3^α2 & β2^, A21^α1 & α3^, H2^α2^ and loop^α5α6^, and A28 loop^α3α4^. It is stabilized by three salt bridges (G3^R43^-H2^D85^, A21^E24^-H2^R64^, and C-terminal A21^T117^-A28^K47^), a π-π stacking (A21^F115^-H2^F176^) and hydrogen bonds (Fig. 3C, dashed lines). The adaptor 2–scaffold 2 interface covers 1402 Å^2^, dominated by contacts involving A21′^α1-α3^ and A28′loops^α3α4 & α4α5^ (Fig. 3D and fig. S9D), along with additional interactions between A21′^α1^, G3′^α2 & β2^ and loop^β2β3^ and H2′^α1 & α2 & α6^ and loop^α5α6^. Four salt bridges (A21′^R28^-A28′^E44^, A21′^D112^-A28′^R57^, A21′^E24^-H2′^R64^, and G3′^K80^-H2′^E189^), one π-cation interaction (A21′^F115^-A28′^K47^), and multiple hydrogen bonds stabilize this interface (Fig. 3D, dashed lines).

Scaffold 2 also forms a unique 787 Å^2^ interface with the central trimer, whereas scaffold 1 does not (Fig. 3E and fig. S9D). The major contacts occur among A16^α7 & α9^ and loop^α6α7^ and G3′^α3^ and L5′ loop^β2α4^, stabilized by a conserved salt bridge (G3′^D99^-A16^R211^) and a network of hydrogen bonds. Additional minor contacts occur among J5^β2^ and G9^β8-β9^ and G3′^β1-β4^, adding further hydrogen bonds (Fig. 3E, dashed lines).

Collectively, approximately 40% of the total EFC surface area participates in intercomponent contacts, underscoring the extensive interaction network critical for EFC assembly. Interfaces rich in electrostatic bonds, particularly between A16-H2′, A21′-H2′, and A21′-A28′, are likely sensitive to protonation and may reorganize under low-pH conditions during membrane fusion. Consistent with this model, prior mutagenesis (*31*, *35*, *39*, *40*, *49*) identified many of these interface residues (table S2 and fig. S9, right, highlighted in red) as critical for VACV infectivity, membrane fusion, and EFC assembly.

### Stabilization and spatial rearrangement of the A16/G9/J5 trimer in the VACV EFC

**Fig. 4.**
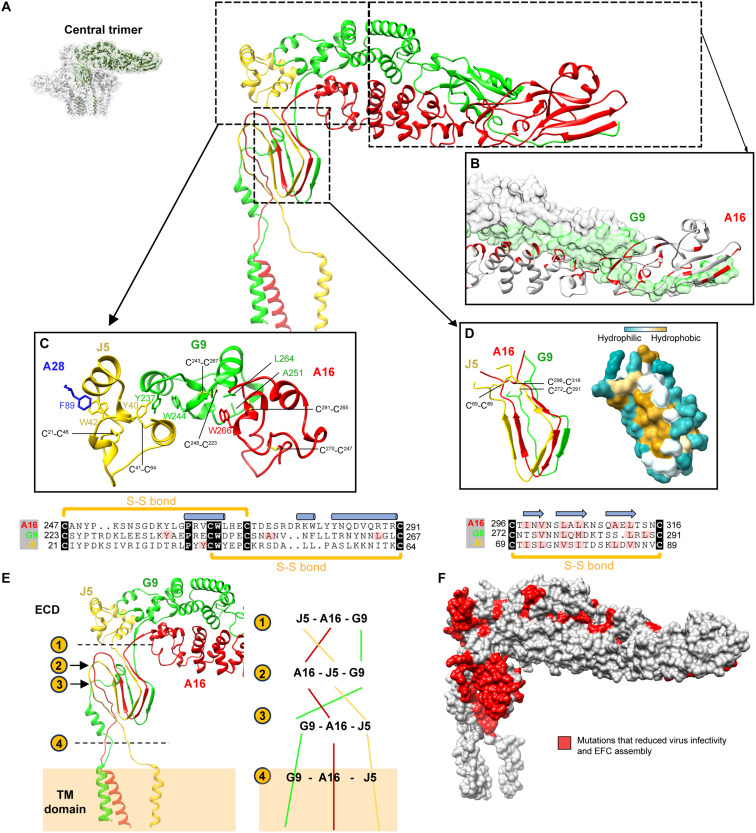
The A16/G9/J5 central trimer is stabilized by conserved structural motifs and intersubunit interfaces. (**A**) Overall architecture of the A16/G9/J5 heterotrimer shown as a cartoon representation. (**B**) Close-up view of the contact interface between the N-terminal domains of A16 (cartoon, red) and G9 (surface, green). Interacting residues are highlighted, and non-interacting regions are shown in gray. (**C**) Trimerization interface formed by conserved PXXCW motifs of A16, G9, and J5. Intermolecular contact residues and disulfide bonds are shown as sticks. Multiple-sequence alignment (MSA) below highlights conserved and contact residues (black and pink shading, respectively), with secondary-structure elements indicated above (helices as columns, β-strands as arrows). (**D**) Close-up of the Delta motifs in A16, G9, and J5, displayed as cartoons with hydrophobic surfaces overlaid. A hydrophobic core is evident at the parallel motif interface. Conserved and contact residues are marked in the accompanying MSA. (**E**) Structural rearrangement of the extended loops linking the ectodomain (ECD) and TM domains of A16, G9, and J5. Four cross-sectional views (left) illustrate the intertwined arrangement of the three subunits, summarized schematically at right. (**F**) Surface representation of the A16/G9/J5 central trimer highlighting residues whose mutations reduce viral infectivity and disrupt EFC assembly (red).

The core structure of the EFC is a heterotrimer formed by A16, G9, and J5 proteins. To dissect the key contacts that stabilize the central trimer, we divided the interface into three regions: top, middle, and bottom (Fig. 4A). The top region (Fig. 4A, right dashed box) is defined by the elongated N-terminal domains of A16 and G9, which are rich in α-helices and β-strands with extensive intermolecular interactions (Fig. 4B and fig. S10). The middle region centers on the conserved PXXCW motif shared by A16, G9, and J5. This motif forms a compact helix bundle stabilized by two disulfide bonds and a conserved tryptophan residue (Fig. 4C). The three motifs of A16, G9, and J5 form a network of hydrophobic and π-π stacking interactions essential for trimer stabilization. For instance, A16 ^W266^ interacts with G9^A251^ and G9^L264^, while G9^W244^ stacks intramolecularly with G9^Y237^, which in turn engages in intersubunit aromatic stacking with J5^Y40^. J5^W42^ further stacks with A28^F89^, linking the central trimer to the A28 adaptor. These conserved aromatic contacts highlight the functional importance of the PXXCW motif in maintaining trimer integrity. The bottom region corresponds to the Delta motif, a ~19-residue segment adopting a triangular strand-loop-strand-loop-strand topology (Fig. 4D and fig. S11). A conserved disulfide bond anchors the termini of the motif, enhancing structural rigidity. The Delta motifs from A16, G9, and J5 are aligned in parallel and form an extended nine-stranded, intermolecular β-sheet, which was not observed in the crystal structure of the truncated A16/G9 complex (*33*) and NMR structure of J5 (*40*). This extensive β-sheet interface formed a buried hydrophobic core (Fig. 4D), which contributes to the tight packing of the central trimer. The central trimer is further stabilized by the intertwining of A16, G9, and J5 proteins (Fig. 4E). As illustrated, these subunits wrap around each other in a braid-like fashion, forming an interlocked complex stabilized primarily by intermolecular hydrogen bonds (fig. S12). The subunit order shifts along the membrane axis: The ectodomains are arranged J5-A16-G9, while the TM helices adopt a G9-A16-J5 pseudo-triangular configuration, suggesting conformational plasticity during EFC assembly and fusion activation. Together, these structural insights of the central trimer reveal previously unrecognized interactions among J5, A16 and G9 through domain-domain interactions and interlocked trimer formation.

Although A16 and G9 form extensive contact across all three regions, earlier biochemical data showed that their N-terminal interactions alone suffice for A16/G9 subcomplex formation in vitro (*33*). Our cryo-EM structure suggests that the PXXCW and the Delta motifs are critical for incorporating J5 into the central trimer. Mutations in either motif disrupt J5 function, reducing virus infectivity and membrane fusion (*40*). Consistently, known function-defective mutations in A16 (*33*), G9 (*33*, *49*), and J5 (*40*) cluster within the trimer interfaces (Fig. 4F and table S2), demonstrating the importance of trimer formation for EFC function.

### The A16 N-domain contains a conserved hydrophobic pocket and phenylalanine-enriched motifs

The cryo-EM structure of the VACV EFC does not directly reveal which component provides the fusogenic domain that inserts into the host membrane to initiate hemifusion. In most viral systems, fusion peptides or loops are short, highly conserved, and enriched in hydrophobic and aromatic residues that mediate membrane insertion (*52*). Several lines of evidence suggest that most EFC proteins are unlikely to serve as direct fusogens. (i) Lipid mixing assays showed that A28, L1, and L5 are dispensable for hemifusion (*53*). (ii) The fusion suppressors A56/K2, expressed at the cell surface, specifically bind A16 and G9 to inhibit cell-cell fusion (*8*, *30*, *42*). (iii) The viral fusion suppressor A26, associated with mature virions, binds A16 and G9 at neutral pH to prevent premature activation (*5*–*7*). (iv) Evolutionary analyses showed that only A16/G9/J5 and F9/L1 homologs are conserved in ancestral giant DNA viruses (*54*). Together, these structural, functional, and phylogenetic observations imply that A16 and G9 as the most plausible fusion protein candidates.

**Fig. 5.**
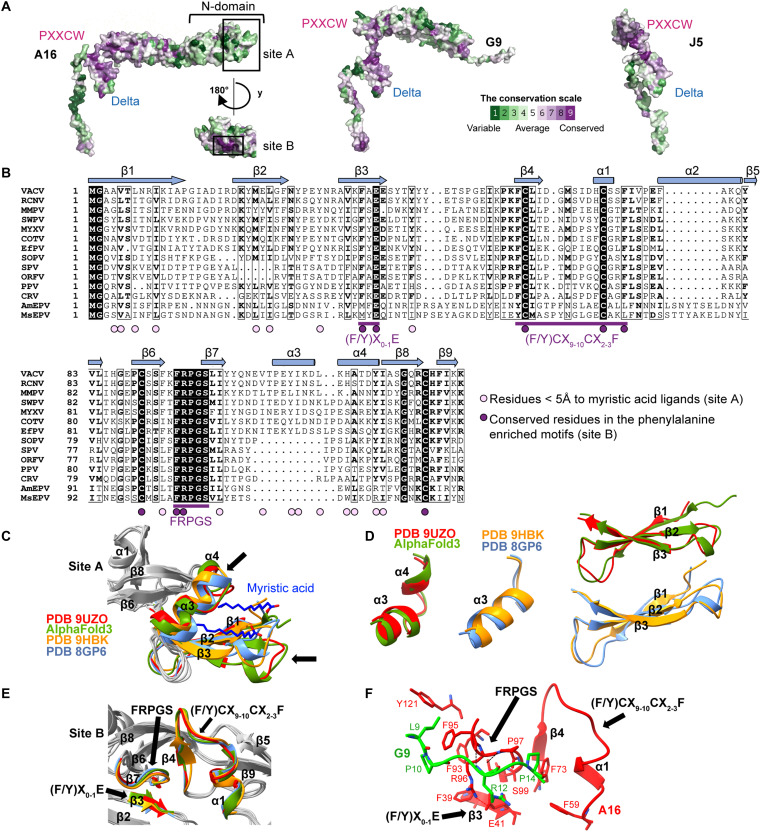
Bioinformatic and structural analyses of the central trimer revealed conserved motifs in the N-terminal domains of A16. (**A**) Surface representations of A16 (left), G9 (middle), and J5 (right) colored by sequence conservation across 52 orthologs from *Poxviridae* family, according to the ConSurf scale (bottom right). Highly conserved residues are shown in purple and variable residues in green. Conserved structural motifs, including PXXCW and Delta, as well as the A16 N-terminal domain (N-domain) containing sites A and B, are labeled. (**B**) Multiple sequence alignment (MSA) of A16 orthologs from 13 representative poxviruses (abbreviations defined in table S3). Three conserved Phenylalanine-rich motifs are underlined. Residues within 5 Å of myristic acid in the AlphaFold model are marked as pink dots, and conserved aromatic residues as purple dots. Secondary structure elements are shown above the alignment (helices as cylinders, β-strands as arrows). (**C**) Structural superposition of the myristoyl-binding pocket among four A16 structures: the EFC-bound cryo-EM structure (PDB 9UZO, red), the AlphaFold3 model (green), the A16/G9-A56/K2 complex (PDB 9HBK, orange), and the A16/G9 crystal structure (PDB 8GP6, light blue). The position of bound myristic acid is shown. Black arrows indicate two regions showing major deviations. (**D**) Close-up view of the lipid-binding cavity showing the deviated α3-α4 and β1-β3 elements from superimposed A16 structures [as in (C)]. (**E**) Structural comparison of site B among the four A16 structures in (C). Black arrows mark the phenylalanine-containing motif. (**F**) Interaction network between the N-terminal domains of A16 and G9 within the EFC structure (PDB 9UZO). The phenylalanine-containing motif of A16 (red) and the contacting residues of G9 (green) are shown as sticks. Hydrogen bonds and salt bridges are indicated by black and red dashed lines, respectively.

To identify potential fusogenic determinants, we performed multiple sequence alignments of 52 members of A16, G9, and J5 orthologs across *Poxviridae* (data S1) and mapped conservation scores from ConSurf (*55*) onto the EFC cryo-EM structure. Two highly conserved surface patches corresponding to the PXXCW and Delta motifs were observed in all three proteins (Fig. 5A), consistent with their central roles in trimerization. In addition, two conserved regions, designated site A and B, were identified at the A16 N terminus (N-domain) (Fig. 5, A and B).

Site A contains a three-stranded β-sheet (β1-β3) and two α-helices (α3 and α4) (Fig. 5, C and D), forming a hydrophobic cavity corresponding to a previously proposed myristoyl-binding pocket (*42*, *56*). Site B, in contrast, is formed by three phenylalanine-enriched motifs, (F/Y)X_0-1_E, (F/Y)CX_9-10_CX_2-3_F, and FRPGS (Fig. 5B). AlphaFold 3 modeling of a myristate-bound A16-G9 complex (ipTM = 0.90 and pTM = 0.89) predicts hydrophobic residues surrounding the ligand within 5 Å (Fig. 5, B and C), suggesting that the pocket’s hydrophobicity, rather than precise residue identity, is sufficient for ligand accommodation across *Poxviridae*. Structural comparison of different A16 conformations revealed that the EFC-A16 closely resembles the AlphaFold 3 myristoylated model but differs from both the x-ray A16 (*33*) and A56/K2-bound A16 cryo-EM structures (Fig. 5C) (*42*). The main differences lie in the helix α3 in the x-ray A16, which are segmented into α3 and α4 in the EFC structure and in the β1-β3 sheet, which shifts slightly downward (Fig. 5, C and D, and fig. S13). These features suggest that the α3-α4 and β1-β3 regions possess conformational flexibility that may facilitate lipid binding.

Site B comprises three highly conserved phenylalanine-enriched motifs: (F/Y)X_0−1_E (residues 39 to 41 of β3); (F/Y)CX_9−10_CX_2−3_F (residues 59 to 73 on β4α1) and FRPGS (residues 95 to 99 on loop^β6β7^) (Fig. 5, B and E). Together, they form a hydrophobic core stabilized by π-π stacking (F39-F93, F59-F73, and F95-Y121) and an electrostatic contact (E41-R96) (Fig. 5F). The FRPGS motif further interacts with the G9 N-terminal loop via hydrophobic contacts (A16^F95^-G9^L9^ and A16^P97^-G9^P14^) and hydrogen bonding (A16^F93^-G9^R12^) (Fig. 5F), reinforcing site B as a conserved hydrophobic core mediating both intra- and intersubunit interactions.

In summary, the A16 N-domain contains a myristoyl-binding pocket (site A) (*42*) and a phenylalanine-rich hydrophobic patch (site B) critical for A16 N-domain folding and its interaction with the G9 N terminus. Inspection of AlphaFold models of A16 orthologs from Myxoma virus, Nile crocodilepox virus, and *Betaentomopoxvirus amoorei*, a continuous hydrophobic surface encompassing site B (fig. S14), demonstrating strong evolutionary conservation of this architecture. Whether these hydrophobic regions become exposed under acidic conditions to mediate membrane fusion remains an important question for future study.

### Interaction of F9 with the EFC

**Fig. 6.**
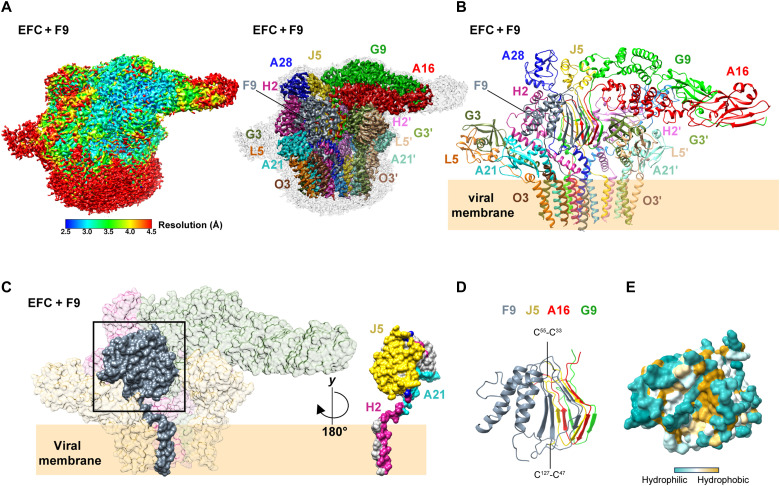
Structure of the EFC + F9 complex and conservation of Delta motifs. (**A**) Cryo-EM density map of the EFC + F9 complex. Left: consensus map colored by local resolution and contoured at 2.5σ. Right: map contoured at 6σ and colored by subunit identity. F9 is shown in gray; EFC components are colored as in Fig. 1C. (**B**) Atomic model of the 16-protein EFC + F9 assembly. Subunits are displayed as ribbons and colored as in (A); the viral membrane boundary is indicated. (**C**) Surface representation of the EFC + F9 complex. Left: overall surface showing the peripheral location of F9 (gray). Right: F9 colored by its interacting partners-J5 (yellow), A21 (cyan), and H2 (magenta), positioning F9 at the membrane-proximal region of the complex. (**D**) Close-up view of the boxed region in (C) showing F9 contact interface with A16/G9/J5 Delta motifs; disulfide bonds are shown as sticks. (**E**) Surface hydrophobicity of the oligomerization interface between F9 and the A16/G9/J5 Delta motifs is colored from hydrophilic (teal) to hydrophobic (tan).

Single-particle cryo-EM reconstruction of the F9-containing complex (EFC + F9) yielded a density map at an overall resolution of 3.05 Å (Fig. 6A, figs. S3 to S5, table S1, and movie S1), contoured at 2.5σ (left) and 6σ (right). The well-resolved map revealed a single F9 molecule peripherally associated with the EFC (Fig. 6B) via multiple contacts: its ectodomain engages J5 and A21, while its TM domain primarily interacts with H2 (Fig. 6C). The F9 protein adopts a compact fold composed of five α-helices packed against a six-stranded β-sheet (Fig. 6D and fig. S6D). Within this architecture, two Delta-like motifs were identified (fig. S11). The first motif (V31-M56) contains a conserved disulfide bond (C33-C55) and closely resembles the topology of the Delta motifs found in A16, G9, and J5. The second motif (V113-T142) lacks this disulfide linkage but retains a similar strand-loop-strand organization. The two Delta-like motifs mediate intermolecular interactions with the J5 Delta motif in the central trimer, forming an extended five-layered β-sheet (Fig. 6D). This inter–β-sheet stacking expands the hydrophobic core shared by J5 and F9 and further stabilizes the overall EFC architecture (Fig. 6E). These findings demonstrate that Delta and Delta-like motifs act as universal β-sheet modules mediating intersubunit stabilization across both central and peripheral assemblies of the EFC.

## DISCUSSION

In this study, we determined the full-length cryo-EM structure of VACV EFC, comprising all 15 component proteins. The EFC adopts a highly organized yet asymmetric architecture centered on a heterotrimer of A16, G9, and J5. This center trimer anchors two A28/H2 adaptor heterodimers, which connect to peripheral scaffolds composed of G3, L5, A21, and O3. Structural alignment with previously solved ectodomain structures revealed minimal deviation, indicating that EFC assembly preserves the ectodomain folds. Notably, the largest interfaces between A16-G9, G3-L5, and A28-H2 correspond to experimentally verified subcomplexes, supporting a hierarchical assembly model driven by thermodynamically favorable interactions (*57*). Conformational variability between the two adaptor-scaffold arms, particularly in their hinge regions, appears to accommodate the asymmetry of the central trimer.

Although the complex was purified from infected cells rather than mature virions, several observations support its assignment as a prefusion state: isolation under neutral pH, and acid-induced conformational transitions that promote liposome association, consistent with prefusion complexes in other viruses (*47*, *48*). The VACV EFC shares neither sequence nor structural similarity with class I-III fusion proteins (*58*–*61*). On the basis of our data, we propose that the EFC defines a distinct class of viral fusion machinery. Central to this system is the A16/G9/J5 trimer, stabilized by the conserved PXXCW and Delta motifs. Gain-of-function mutations in G9 (H44Y, H44R, and Y42C) (*62*, *63*) enhance fusion without disrupting assembly, underscoring their direct role in fusogenic regulation. Most notably, the N terminus of A16 contains two hydrophobic regions, site A and site B, that contribute to its structural stability and interaction with G9. Site A forms a hydrophobic pocket consistent with a previously proposed myristoyl-binding site (*42*, *56*). This pocket, although compatible with lipid accommodation, shows limited sequence conservation among *Poxviridae*, suggesting a structural role in positioning or shielding the N-terminal myristoyl groups of A16 and G9 rather than acting as the fusion peptide itself. Site B contains a conserved phenylalanine-rich aromatic cluster that stabilizes the A16 N-domain fold and mediates hydrophobic contact with G9. In the prefusion structure, this region is masked by the G9 N-terminal loop, consistent with a closed configuration that conceals the myristates. Thus, the conserved hydrophobic surface of A16 primarily stabilizes the A16-G9 interface and regulates myristate positioning, while its potential rearrangement during activation remains to be determined. Capturing the EFC structure under acidic conditions will be crucial for defining the conformational transitions and catalytic residues that mediate membrane insertion. Future cryo-EM studies under acidic conditions will be crucial for visualizing these structural transitions and determining how the A16 N-terminal hydrophobic region interacts with the host membrane during fusion.

F9 is conserved across the *Poxviridae* and exhibits sequence and structural similarity to L1 but lacks N-terminal myristoylation. (*29*, *36*, *37*). Genetic and cell-based studies indicate that F9 is essential for virus infectivity and for low-pH-triggered cell-cell fusion, whereas the EFC can assemble in the absence of F9 (*29*, *36*). In our EFC + F9 cryo-EM reconstruction, F9 engages the complex through ectodomain interfaces with J5 and A21 and a membrane-proximal contact with H2, positioning F9 at the membrane-proximal region that could influence conformational transitions near the membrane, consistent with the previous observation that three EFC components A21, H2, and F9 are required for the hemifusion step (*53*). These observations support a model in which F9 modulates EFC function, potentially tuning hemifusion or fusion efficiency, rather than acting as a central assembly determinant. The only known EFC component absent from our cryo-EM reconstruction is L1. The lack of L1 density, together with the predicted steric clash between L1 and F9 observed in AlphaFold modeling (*41*), raising the possibility that F9 and L1 associate with the EFC in a mutually exclusive manner. Defining how L1 engages the EFC and whether F9 gates specific fusion steps will require time-resolved structural analyses in the future.

As mentioned in the Introduction, VACV mature virions contain two fusion inhibitors, A26 and A56/K2, that regulate the activity of the EFC. A26 performs dual roles during MV entry: It mediates viral attachment to cell-surface laminin and restrains EFC-mediated membrane fusion until triggered by low pH during endocytosis. Structural and mutational analyses have shown that two histidines in the N-terminal region of A26 (H48 and H53) are critical for this process (*7*). Upon protonation at low pH, these residues induce conformational rearrangements in A26 that weaken its interaction with the A16/G9 subcomplex. This conformational change acts as a mechanical trigger, transmitting the pH signal from the environment to the EFC and initiating a cascade of structural rearrangements within EFC proteins that ultimately promote membrane fusion. We therefore view A26 as an acid-sensitive fusion suppressor that couples environmental pH sensing to EFC activation. In the presence of A26, the EFC is maintained in a prefusion-locked conformation, preventing premature fusion during virion assembly and release. At acidic pH, A26 undergoes a conformational change and dissociates from A16/G9, relieving this suppression and allowing the EFC to transition toward its fusion-active state. In contrast, when A26 is deleted, MVs can enter cells directly at the plasma membrane under neutral pH conditions, consistent with an EFC that assembles in a primed or partially activated configuration in the absence of A26 during morphogenesis. Moreover, in cells coexpressing A26, A56/K2, and A16/G9 together, coimmunoprecipitation analysis detected A16/G9 associated with either A26 or A56/K2, but not both, suggesting that A26 and A56/K2 engage overlapping binding sites on A16/G9 (*6*). This model reconciles the ability of VACV to use both pH-dependent and pH-independent entry pathways. Acidic pH does not act directly on the EFC but instead regulates conformational changes in the fusion regulators A26 and A56/K2, which in turn transmit mechanical forces to the EFC to trigger its structural transitions. The resulting remodeling of A16/G9 and neighboring components likely exposes the hydrophobic N-terminal region of A16, enabling its insertion into the host membrane and initiating membrane fusion. Recently, the cryo-EM structure of the A16/19 subcomplex bound to the fusion suppressor A56/K2 was reported (*42*). Future determination of the structure of A16/G9 bound to A26 will clarify whether both fusion suppressors target the same region of A16/G9 and share a common mechanism of fusion regulation.

AlphaFold modeling has become a widely adopted approach for predicting unknown protein structures, achieving remarkable accuracy for single-protein models (*64*). However, reliable prediction of multimeric complexes often requires additional structural restraints or complementary experimental data (*65*). Predicted structures of the G3/L5/A21/O3 subcomplex (*38*) closely match our experimentally determined scaffold module, confirming the accuracy of computational predictions when correct stoichiometry is applied. A separate AlphaFold model proposed a 10-subunit EFC, which, although differing from the cryo-EM structure in stoichiometry, correctly predicted the parallel alignment of A16, G9, and J5 Delta motifs, reflecting the inherent trimer organization (*41*). When the appropriate stoichiometry is imposed, AlphaFold predicted a three-layer arrangement (fig. S15) similar to our experimental model. The most notable structural deviation lies in the orientation of the A16/G9 ectodomains, which, in the cryo-EM structure, are positioned in parallel to the viral membrane, whereas in the AlphaFold model, the N terminus of A16/G9 points away from the viral membrane. Whether this represents an incorrect model or an alternate energy state, such as the low-pH form, will require further investigation. Experimental visualization of the EFC under fusogenic conditions will be critical to distinguishing between these possibilities.

In summary, our results establish the VACV EFC as a unique class of multiprotein fusion machinery, distinct from the canonical classes I, II, and III systems. The cryo-EM structure defines the complete architectural framework, reveals the interlocking A16/G9/J5 trimer stabilized by PXXCW and Delta motifs, and uncovers conserved hydrophobic modules in A16 that may regulate lipid engagement. Future work capturing acidic or intermediate conformations will be critical to elucidate EFC-mediated membrane fusion during poxvirus entry.

## MATERIALS AND METHODS

### Cells, viruses, chemicals, and reagents

HeLa cells were cultured in suspension in Dulbecco’s modified Eagle’s medium (DMEM; Gibco) supplemented with 10% fetal bovine serum (FBS; Cytiva Inc.). The recombinant VACV vA28-SBP is derived from the wild-type WR strain. The nomenclatures of VACV EFC proteins follows the Copenhagen strain, with the corresponding orthopoxvirus gene (OPG) identifiers provided in parentheses as follows: A16L (OPG143), G9R (OPG094), J5L (OPG104), A28L (OPG155), H2R (OPG107), G3L (OPG086), L5R (OPG099), A21L (OPG147), O3L (OPG076), F9L (OPG053), and L1R (OPG095). Antibodies recognizing VACV EFC proteins were described previously (*35*, *54*). All chemicals for preparing liposomes were obtained from Sigma-Aldrich unless otherwise specified. 1-Palmitoyl-2-oleoyl-glycero-3-phosphocholine (POPC), 1-palmitoyl-2-oleoyl-sn-glycero-3-phosphoethanolamine (POPE), brain sphingomyelin (SM), and bis(monooleoylglycero)phosphate (S,R isomer) (ammonium salt) (BMP) were purchased from Avanti Polar Lipids (Alabaster, AL, USA).

### Generation of A28-SBP tagged recombinant VACV

The vA28-SBP virus was derived from the vA28-HAi virus (*21*) by inserting an SBP (sequence: MDEKTTGWRGGHVVEGLAGELEQLRARLEHHPQGQREP) at the C terminus of A28L. To generate vA28-SBP, an A28L transfer plasmid was constructed by assembling three DNA fragments, the A28-SBP fragment, the A29L gene, and the A27 gene cassette, into a pCRII-TOPO plasmid. Detailed cloning procedures are described below: First, the full-length A29L gene was polymerase chain reaction (PCR) amplified from vA28-HAi genomic DNA using the following primers: primer a, 5′-AAAAAGCTTATGCAGCATCCGCGGGAAG-3′ (Hind III site underlined); and primer b, AAAGGATCCTTATAATCTATTAGAAGCTGAC-3′ (Bam HI site underlined). To obtain the A28L-SBP fragment, the A28L gene (under a p11k promoter) and the SBP-tag were amplified from the vA28-HAi genome and a SBP tag–containing plasmid (pCRII-TOPO-G2/iG3-CTAP/G1), respectively, using the following primers: For the p11k/A28L fragment: primer c, AAAGGATCCGAATTTCATTTTGTTTTTTTCTATGC (Bam HI underlined); and primer d, AAAGCTCTTCATCCAAGTACAGATTTTAGAAACTGAC (Sap I underlined). For the SBP tag: primer e, AAAGCTCTTCA*GGATCTTGTTGTCCTGGCTGTTGCGGATCT*ATGGACGAGAAGACCACC (Sap I underlined; GS-tetracysteine-GS linker in italics); and primer f, AAACTCGAGTTAAGGCTCGCGTTGCCCCT (Xho I underlined). These two fragments were ligated together. The A27L gene cassette was PCR amplified from vA28-HAi genomic DNA. Because insertion of the SBP tag at the 3′end of A28L disrupted the native A27L promoter, a synthetic early/late promoter was fused upstream of A27L to drive its expression. The following primers were used: primer g, AAACTCGAG*CCAAAAAATTGAAATTTTATTTTTTTTTTTTGGAATATAA*ATGGACGGAACTCTTTTCC (Xho I underlined; promoter in italics); and primer h, AAATCTAGATTATAAAATCGTAGATCTCCCATG (Xba I underlined). All fragments were digested with the appropriate restriction enzymes and ligated into Hind III– and Xba I–linearized pCRII-TOPO vector at a 1:1 molar ratio, yielding the final pCRII-TOPO-A29/A28L-SBP-A27 construct. The construct was sequence verified to confirm the absence of mutations. Recombinant virus vA28-SBP was generated on the basis of established infection-transfection techniques described previously (*66*). In brief, CV-1 cells were infected with vA28-HAi at a multiplicity of infection (MOI) of 1 PFU per cell in the absence of IPTG, and transfected with 1 μg of pCRII-TOPO-A29/A28L-SBP-A27 plasmid. Homologous recombination replaced the GUS/PT7/lacO/A28-HA cassette in the vA28-HAi genome. Cells were harvested 48 hours postinfection (hpi) and used for clonal isolation of recombinant vA28-SBP virus after four rounds of plaque purifications.

### Isolation and purification of VACV EFC

HeLa cells were cultured in suspension in DMEM supplemented with 10% FBS, penicillin (100 U/ml), and streptomycin (100 μg/ml; Invitrogen) with continuous mixing using a magnetic stir bar at 100 rpm at 37°C. Cells (2 × 10^6^ cells/ml) were subsequently infected with a recombinant VACV vA28-SBP at a MOI of 1 PFU per cell and continued cultured at 37°C until being harvested at 24 hpi. Cells then were centrifuged at 2000*g* for 10 min at 4°C to collect cell pellets. Cell pellets from 1 liter of culture were resuspended and lysed in 50 ml of lysis buffer containing 0.5% NP-40, 200 mM NaCl, 20 mM tris-HCl (pH 8.0), 1 mM EDTA, deoxyribonuclease (2 μg/ml), and a protease inhibitor cocktail [aprotinin (2 μg/ml), leupeptin (1 μg/ml), pepstatin A (0.7 μg/ml), and 1 mM phenylmethylsulfonyl fluoride]. Lysis was carried out at 4°C with gentle mixing (15 rpm) for 1 hour. Lysates were cleared by centrifugation at 15,000*g* for 1 hour at 4°C using a JLA-16.250 rotor (Beckman Coulter). The resulting supernatant was sequentially filtered through 0.45- and 0.2-μm syringe filters (Minisart, Sartorius). The clarified lysate was loaded onto a 1 ml of HiTrap Streptavidin HP column (Cytiva Inc.) pre-equilibrated in lysis buffer. The column was washed sequentially with 20 column volumes each of the following four buffers: (i) lysis buffer, (ii) washing buffer 1 [0.5% *n*-dodecyl β-d-maltoside and 0.02% NP-40 in phosphate-buffered saline (PBS)], (iii) washing buffer 2 (0.02% NP-40 in PBS with 300 mM NaCl), and (iv) washing buffer 3 (0.02% NP-40 in PBS). Bound EFC was eluted with PBS containing 2.5 mM biotin and 0.02% NP-40. The purified EFC was used immediately for cryo-EM sample preparation.

### Liposome preparation and biochemical analyses

Liposomes were prepared using the thin-film hydration method followed by freeze-thaw cycling and membrane extrusion (*67*). A lipid mixture consisting of POPC, POPE, brain SM, cholesterol, and BMP in a molar ratio of 30:15:15:15:25 was dissolved in chloroform. The solvent was removed by rotary evaporation to form a thin lipid film and dried overnight using a freeze dryer. The dried lipid film (17.6 μmol total lipids) was hydrated with 1 ml of PBS (pH 7.4). The resulting lipid suspension was subjected to more than 10 freeze-thaw cycles using alternating immersion in liquid nitrogen and a 50°C water bath. The suspension was then extruded 21 times through 50-nm-pore-size polycarbonate membranes (Avanti Polar Lipids) using a Mini Extruder (Avanti Polar Lipids) at 50°C to yield uniform unilamellar vesicles.

Phospholipid concentration was quantified using a modified ascorbic acid–based colorimetric assay (*68*), to determine the lipid concentration of liposomes. In brief, 0.45 ml of 8.9 N H_2_SO_4_ was added to phosphate standards (NaH_2_PO_4_, 32.5 to 227.5 nmol) and liposome samples (15 or 30 μl). The tubes were heated at >200°C for 25 min, then cooled for 5 min at room temperature. Subsequently, 0.15 ml of 10% H_2_O_2_ was added to each tube, followed by a second heating step at >200°C for 30 min. After cooling, 3.9 ml of deionized water, 0.5 ml of 2.5% (w/v) ammonium molybdate tetrahydrate, and 0.5 ml of 10% (w/v) ascorbic acid were sequentially added. The reaction mixtures were incubated at 100°C for 7 min. After cooling, absorbance was measured at 820 nm using a Varian Cary 50 Scan UV-Visible spectrophotometer (Agilent). Phospholipid concentration was calculated using standard calibration curve of phosphate standards.

Lipid composition of liposome in different centrifugation fraction was checked by thin-layer chromatography (TLC) to make sure no major lipid composition difference in different fractions. To remove most of the iodixanol (gradient centrifugation additives) and to intensify lipid signal, 150 μl of liposome solution was mixed with 150 μl of chloroform and 300 μl of methanol (*69*), followed by vortexing for 2 min. Subsequently, 150 μl more chloroform was added and vortexed for 30 s, followed by the addition of 150 μl of deionized water and another 30 s of vortexing. After phase separation, the upper aqueous layer was discarded, and the lower organic phase was collected and dried at 150°C. The dried lipids were resuspended in 40 μl of chloroform. The lipid solution was spotted onto a silica gel TLC plate. Lipid separation was performed using a developing solvent composed of chloroform:methanol:water (70:25:4, v/v/v) (*70*). After development, lipids were visualized by staining with iodine vapor.

Dynamic light scattering measurements were performed using a Zetasizer Nano ZS instrument (Malvern, United Kingdom) equipped with a 633 nm He-Ne laser. Liposome samples were diluted to a final concentration of 50 μM lipid in PBS, pH 7.4. The size distribution, zeta potential, and particle concentration of liposome solutions were measured at 25°C.

To visualize the size distribution of the liposomes, cryo-EM grids (200-mesh copper, HC200-Cu, PELCO) were glow-discharged for 15 s on the carbon-coated side in an argon/oxygen atmosphere. A 4-μl aliquot of liposome solution (final lipid concentration: 0.7 mM) was applied to each grid. Grids were blotted for 3 to 4 s at 100% humidity and 4°C, then plunge-frozen into liquid ethane cooled by liquid nitrogen using a Vitrobot (FEI, Hillsboro, OR). Samples were imaged using an FEI Tecnai G2 F20 TWIN transmission electron microscope operated at 200 keV in bright-field mode at the Cryo-EM Facility of Academia Sinica (Taipei, Taiwan). Images were acquired under low-dose conditions (25 to 30 e^−^/Å^2^) at a nominal defocus of ~1.8 μm using a 4 k by 4 k charge-coupled device camera (Gatan, Pleasanton, CA) at 50,000× magnification.

### Protein-liposome interaction assays

Protein-liposome interaction assays were conducted as previously described (*48*). Purified EFC protein was incubated with preformed liposomes at a 1:600 protein-to-lipid molar ratio at 37°C for 5 min. To induce acid-dependent conformational changes, the pH was adjusted to 6, 5, and 4 by adding 0.1 M citric acid–sodium citrate buffer. Following a 2-hour incubation at 37°C, the mixture was neutralized to pH 7.0 using 2 M tris-HCl (pH 9.0). OptiPrep density gradient medium (Sigma-Aldrich) was added to a final concentration of 36%, with 0.15 M NaCl maintained throughout. A 167-μl aliquot of the sample was transferred to an open-top Thickwall polycarbonate tube (Beckman Coulter) and overlaid with 835 μl of 30% OptiPrep containing 0.15 M NaCl. Samples were centrifuged at 57,344*g* for 70 min at 4°C using a TLA120.2 rotor (Beckman Coulter). Fractions were collected sequentially from the top to the bottom of the tube and analyzed by immunoblotting to determine the distribution of EFC components.

### Cryo-EM sample preparation

Purified EFC sample (4 μl) was applied to glow-discharged Quantifoil R1.2/1.3 holey carbon grids (Quantifoil GmbH) coated with either a 2-nm continuous carbon film or graphene oxide (GO). Grids were vitrified using a Vitrobot Mark IV (Thermo Fisher Scientific) at 4°C and 100% relative humidity. After a 10 s waiting time, excess liquid was blotted for 3.5 to 4.0 s using filter paper, and the grids were rapidly plunged into liquid ethane precooled with liquid nitrogen. Grids were stored in liquid nitrogen until further use.

### Cryo-EM data acquisition

Grids were initially screened using a Talos Arctica transmission electron microscope (Thermo Fisher Scientific) operating at 200 kV and equipped with a Falcon III direct electron detector in linear mode. Images were acquired at a nominal magnification of 120,000×, corresponding to a calibrated pixel size of 0.86 Å/pixel, with a defocus setting of −3.0 μm. For high-resolution data collection, selected grids were transferred to a Titan Krios G3i transmission electron microscope (Thermo Fisher Scientific) operating at 300 kV and equipped with an X-FEG electron source, a BioQuantum energy filter (15 eV slit width; Gatan), and a K3 Summit direct electron detector (Gatan) operating in super-resolution mode. Automated data acquisition was performed using EPU software (version 3.6; Thermo Fisher Scientific) at a nominal magnification of 81,000×, yielding a physical pixel size of 0.5305 Å/pixel. The defocus range was set from −1.4 to −2.4 μm. Each movie stack consisted of 50 frames saved as non–gain-normalized tagged image file format (TIFFs), with a total exposure time of 1.34 s, corresponding to a dose rate of ~37.3e^−^/Å^2^/s and a total accumulated dose of ~50.4e^−^/Å^2^ (~1e^−^/Å^2^ per frame). Cryo-EM data acquisition parameters are summarized in table S1.

### Single-particle image processing and 3D reconstruction

A total of 17,298 movie stacks were processed, including 8064 acquired from GO-coated grids and 9234 from 2-nm-thin carbon-coated grids, all collected in super-resolution mode. Motion correction and dose weighting were performed using MotionCor2 (*71*) with a 16 by 11 patch size and no binning, resulting in a final pixel size of 0.5305 Å/pixel. Contrast transfer function (CTF) parameters were estimated from motion-corrected, dose-weighted micrographs using CTFFind4 (*72*) implemented within cryoSPARC (*73*). Particles were automatically picked in cryoSPARC using 2D templates derived from an initial model generated from the Talos Arctica dataset. Picked particles were extracted in 640-pixel boxes and down sized to 128 pixels to accelerate processing. Following 2D classification to remove poor class averages, the remaining particles (3,816,837 from the GO dataset and 5,229,620 from the thin-carbon dataset) were subjected to ab initio reconstruction and heterogeneous refinement without imposing any symmetry (C1). After three rounds of heterogeneous refinement (C1) followed by 2D classification, particles belonging to poorly defined classes were discarded. A subset of particles (431,035 from the GO dataset and 720,975 from the thin-carbon dataset) exhibiting better features in one 3D class was selected and extracted with the original box size of 640 pixels for further refinement. Another round of ab initio reconstruction and heterogeneous refinement was conducted without applying symmetry (C1). The combined pool of high-quality particles (1,001,279) from both datasets was then refined using nonuniform refinement without symmetry (C1), yielding a 3D reconstruction with a resolution of 2.91 Å.

To investigate heterogeneity or dynamics within the reconstruction, 3D variability analysis was performed, revealing the presence of the F9 subunit as the most notable difference. Subsequently, 3D classification focused on the F9 subunit region was carried out, resulting in two EFC complex structures: one without (EFC) and one with the F9 subunit (EFC + F9). These two structures were further refined using nonuniform refinement without symmetry (C1), achieving resolutions of 2.98 (EFC) and 3.05 Å (EFC + F9), respectively. Map sharpening and resolution estimation were performed using cryoSPARC (*73*). The overall resolution was determined on the basis of the Fourier shell correlation at 0.143, and local resolution was also calculated within cryoSPARC. The resulting 3D density maps were visualized using UCSF Chimera (*74*) or UCSF ChimeraX (*75*). The detailed procedures for single-particle image processing are illustrated in fig. S3, while the specifics of the cryo-EM reconstruction are summarized in figs. S4 and S5. Additional statistical information regarding the cryo-EM reconstructions is presented in table S1.

### Structure determination and model building

For atomic model building of the cryo-EM map, we first used AlphaFold3 (*76*) to predict the structure of the entire complex, containing one copy of each subunit. After obtaining the cryo-EM structure of the EFC complex, we attempted to fit the AlphaFold-predicted model of the whole complex into the cryo-EM density map; however, the overall fit was poor, likely due to conformational differences and the unknown stoichiometric ratios among the subunits. Therefore, we instead fitted the individually predicted structures of each subunit into the cryo-EM map one by one. Regions with poor fitting or conformational discrepancies were manually adjusted using COOT (*77*). Disordered C-terminal loops of A16, G9, and J5; the TM domains of both copies of G3/G3′ and L5/L5′; and the A16 N-terminal domain (residues 8 to 41 and 109 to 121) were deleted and manually rebuilt in COOT. For the EFC + F9 complex, the map was modeled following the same procedure as for the EFC complex. The additional density observed in the EFC + F9 map was modeled using one molecule of the AlphaFold3-predicted F9 subunit. The complete model was further refined in PHENIX (*78*) using the real-space refinement module with default parameters. As a result, we obtained an atomic model of the entire complex with corrected subunit conformations and stoichiometry, comprising one each of A16, G9, and J5 subunits; two each of A21, A28, G3, H2, L5, and O3 subunits for the EFC complex without F9; and an additional F9 subunit for the EFC + F9 complex. Last, the model was validated using the Comprehensive validation (cryo-EM) tool implemented in PHENIX (*78*).

### A16/G9/J5 sequence conservation analysis

Homologous sequences of A16, G9, and J5 from members of the *Poxviridae* family were extracted from a previous study (*54*) and are detailed in data S1. We identified a total of 52 homologs for A16, G9, and J5 proteins. The percentage identity of each ortholog group compared to its query sequence ranges from 19 to 99.7% for A16 orthologs, from 20 to 99.7% for G9 homologs, and from 20 to 98.5% for J5 homologs. Multiple sequence alignment analyses for each ortholog group were performed using Clustal Omega (*79*). The aligned sequences were then visualized using ENDscript (https://espript.ibcp.fr/ESPript/ESPript/) (*80*). The conservation of amino acids at each site was calculated using the Rate4Site program in ConSurf (*81*, *82*). This analysis generates a total of nine groups of scores, which reflect the evolutionary rate of each amino acid site. A high score indicates slowly evolving amino acids, which are considered conserved residues, while a low score indicates rapidly evolving amino acids, classified as variable.

### AlphaFold model prediction

We used both AlphaFold 3.0 (https://alphafoldserver.com) and ColabFold version 1.5.5 (https://colab.research.google.com/github/sokrypton/ColabFold/blob/main/AlphaFold2.ipynb) to model *Poxviridae* homologs of A16 and A16-G9 heterodimer complexes. The predicted models were evaluated only when the residue pLDDT was greater than 70. The overall structure pTM exceeded 0.65, except 0.54 for Nile crocodile poxvirus and 0.56 for Betaentomopoxvirus amoorei. For protein complexes, the iPTM scores were required to be above 0.7.

### Use of artificial intelligence (AI)

AI-assisted language editing tool ChatGPT 5.2 was used exclusively to improve the accuracy of the text and correct grammatical errors. Text segments were submitted to the model alongside the following instructional prompts: “Edit the following paragraph for grammar errors,” “Edit the following paragraph to improve the English clarity,” “Polish the English for accuracy,” “Edit the text to fix grammar errors,” “Edit the English,” “Edit text for errors,” “Polish the following text,” and “Edit the text to improve readability.” All generated suggestions were evaluated manually; only valid corrections were accepted and incorporated into the final text. These tools did not generate scientific content, figures, references, or interpretations. The authors take full responsibility for the content of the published article.
