# Supplementary material for: Cryo-EM structure of the vaccinia virus entry fusion complex reveals a multicomponent fusion machinery
Source: Sci Adv. 2026 Jan 14;12(3):eaec0254. doi: 10.1126/sciadv.aec0254 (PMC12802840; doi:10.1126/sciadv.aec0254)
Supplement: Supplementary file 1 — Figs. S1 to S15 Tables S1 to S3 Legend for movie S1 Legend for data S1 [file sciadv.aec0254_sm.pdf]

Science Advances
AAAS

Supplementary Materials for

Cryo-EM structure of the vaccinia virus entry fusion complex reveals a multicomponent fusion machinery

Chang Sheng-Huei Lin *et al.*

Corresponding author: Chang Sheng-Huei Lin, lin77tw@as.edu.tw; Wen Chang, wenchang@as.edu.tw

Sci. Adv. **12**, eaec0254 (2026)
DOI: 10.1126/sciadv.aec0254

The PDF file includes:

Figs. S1 to S15
Tables S1 to S3
Legend for movie S1
Legend for data S1

Other Supplementary Material for this manuscript includes the following:

Movie S1
Data S1

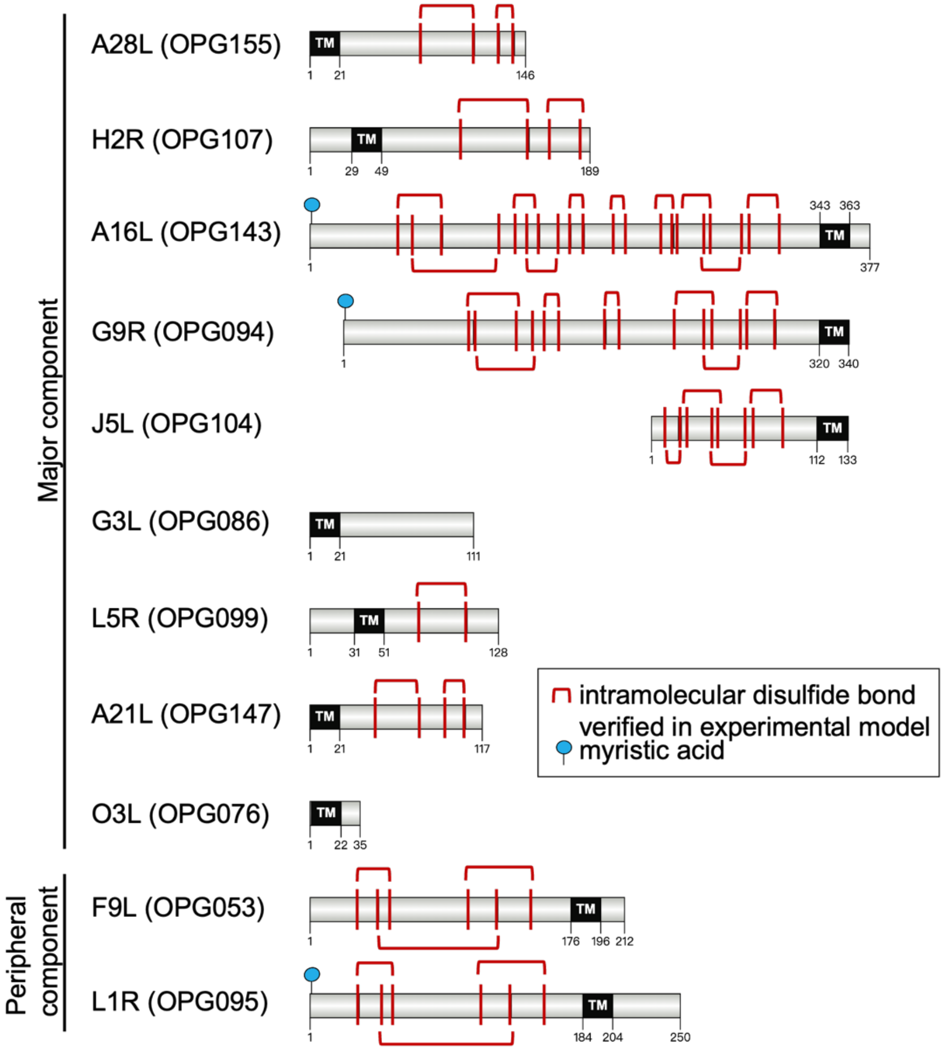

Fig. S1.
Schematic diagram of 11 VACV EFC protein components. Predicted TM domains are shown as black boxes. Intramolecular disulfide bonds are connected with red lines. N-terminal myristoylation sites are marked as filled circles in cyan. The orthopoxvirus gene (OPG) names for each VACV EFC component are indicated in the brackets.

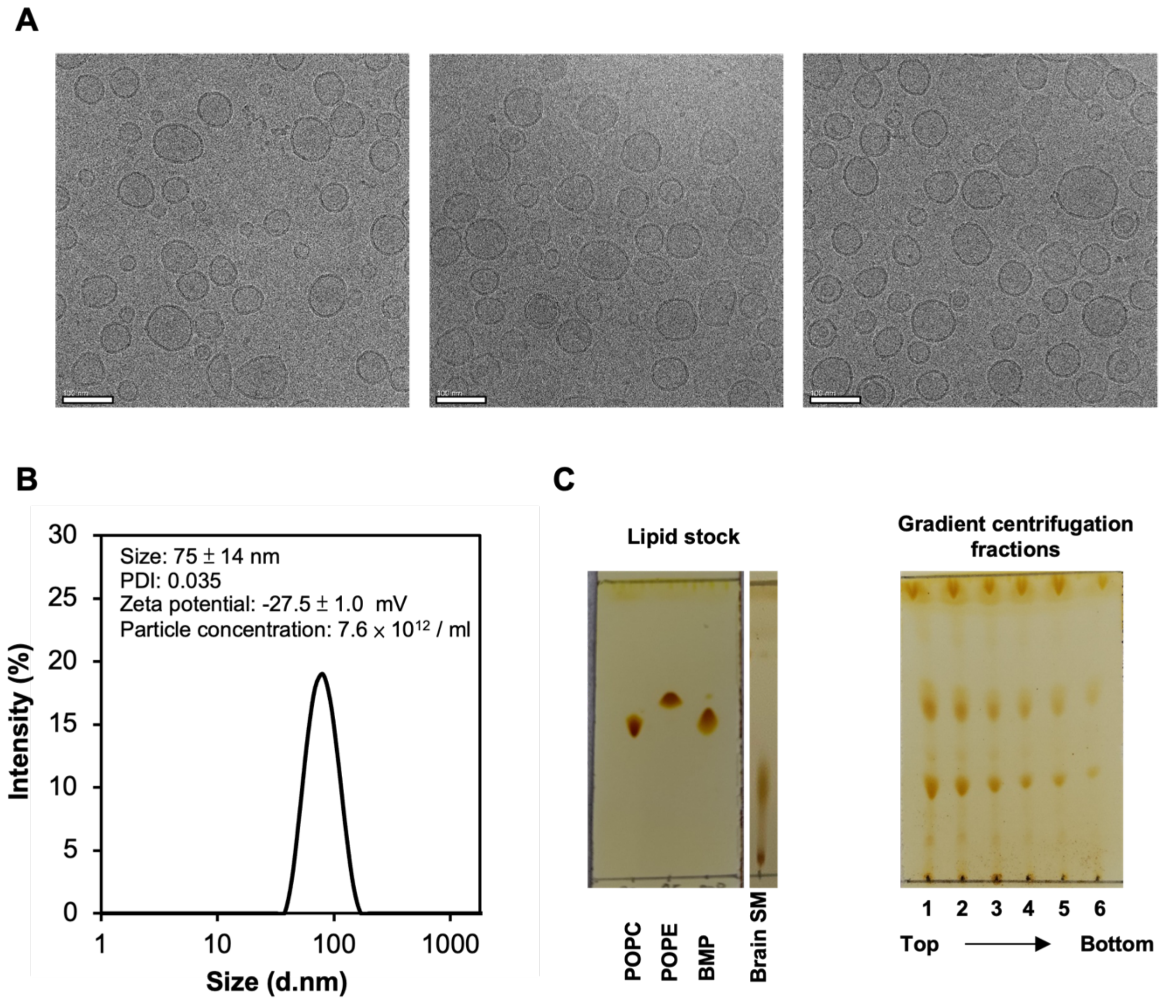

Fig. S2.

Biophysical and biochemical characterization of synthesized liposomes. (A) Representative cryo-EM micrographs of the liposomes at a magnification of 50,000× (triplicate). Scale bar: 100 nm. (B) Size, zeta potential, and particle concentration of the liposomes. (C) TLC of single pure lipids (left) and gradient centrifugation fractions (right, lanes 1 to 6). Collected fractions were analyzed by TLC with resolving solution (chloroform: methanol: water = 70:25:4, v/v/v) and showed that 70% of liposomes became localized in fractions 1-3, indicating their floating capacity.

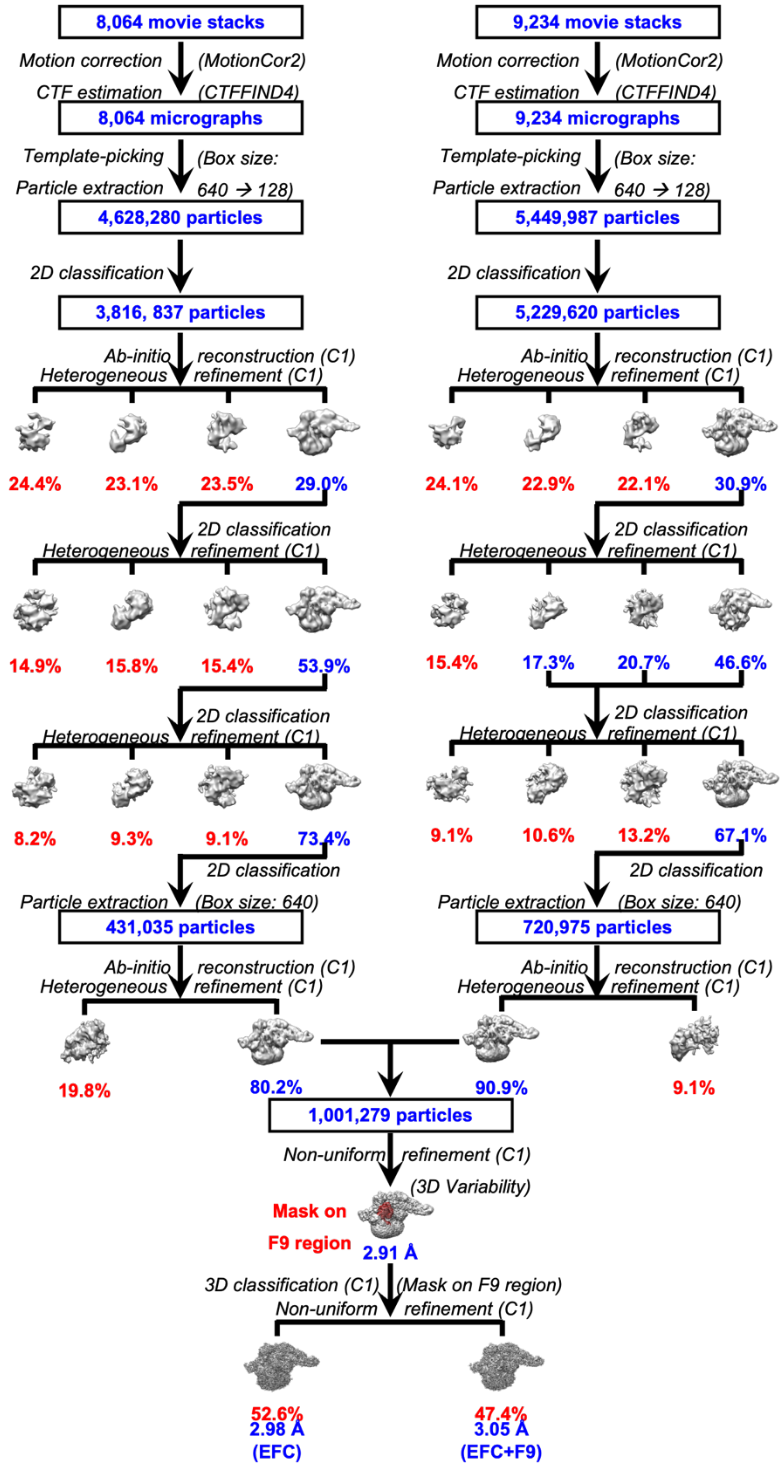

Fig. S3.

Data-processing workflow for cryo-EM reconstruction of the EFC and EFC+F9 complexes. Two cryo-EM datasets were collected and subjected to motion correction and CTF estimation to compensate for beam-induced motion and determine CTF parameters. Particle picking was performed using a template-based approach, followed by 2D classification to remove low-quality

particles and ice contaminants. *Ab initio* reconstruction was used to generate initial 3D maps of the EFC complex, which were subsequently refined through three rounds of 3D heterogeneous refinement, and an additional round of 2D classification was performed to select high-quality particles further. After particle re-extraction to the original box size, *ab initio* reconstruction and 3D heterogeneous refinement were repeated to remove remaining suboptimal particles. The final selected particles from both datasets were merged and refined using non-uniform refinement, yielding a consensus EFC map at 2.91Å resolution. Three-dimensional variability analysis revealed two distinct conformations of the EFC, one with and one without the F9 subunit. Focused 3D classification using a mask on the F9 subunit region was subsequently performed. Final non-uniform refinement of each subset produced high-resolution maps of the EFC (2.98Å) and EFC+F9 (3.05Å). Additional details of image processing and refinement parameters are provided in the **Methods** section.

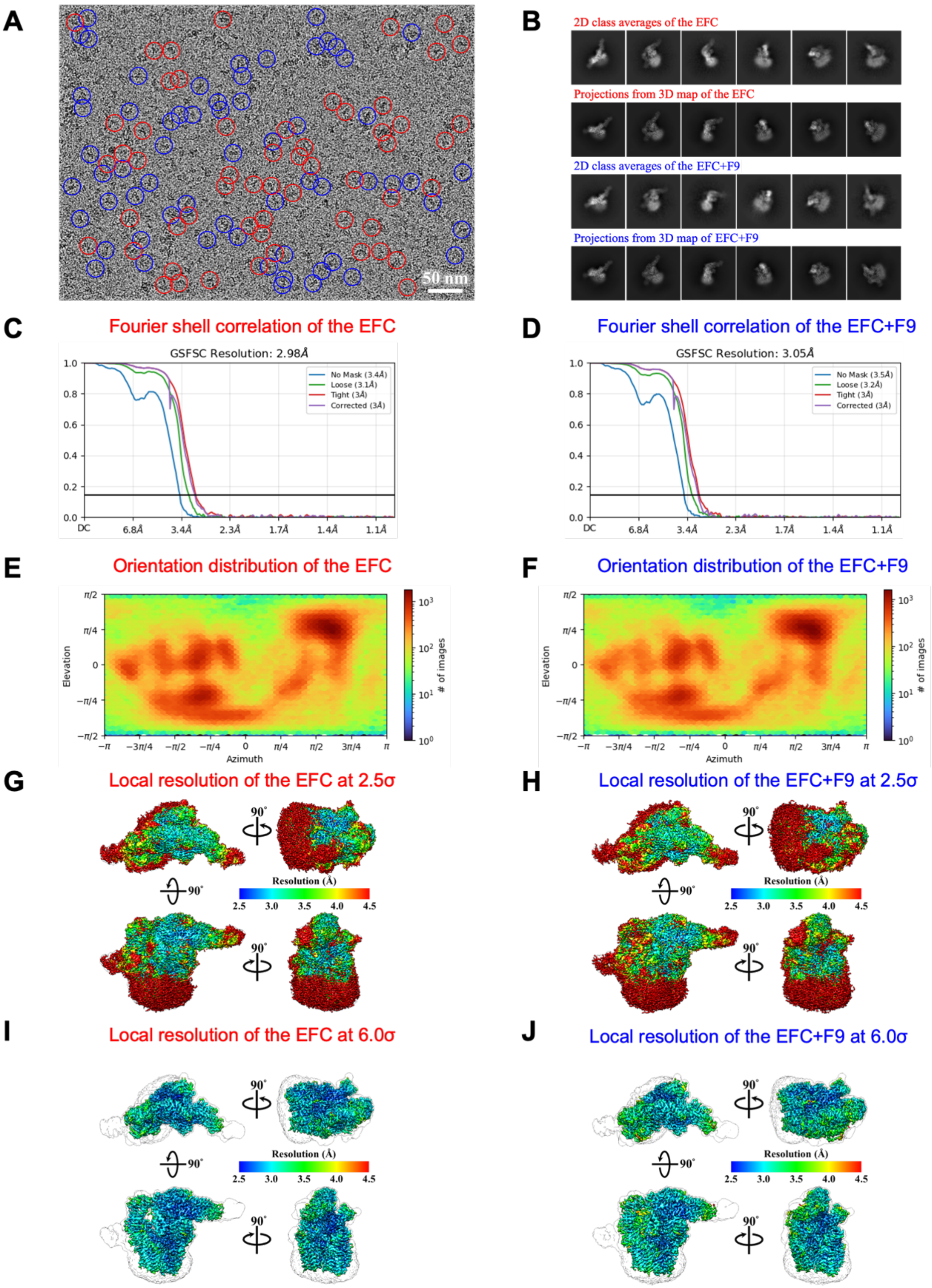

Fig. S4.

Cryo-EM structure determination of the EFC complexes. (A) Representative motion-corrected cryo-EM micrographs. Particles selected for final reconstruction are marked with red circles (EFC) and blue circles (EFC+F9). (B) Representative 2D class averages, demonstrating particle quality and orientations. Corresponding 3D map projections in similar orientations are

also shown for comparison. (C) Gold-standard FSC curve (threshold at FSC = 0.143) for the EFC. (D) Gold-standard FSC curve (threshold at FSC = 0.143) for the EFC+F9. (E) Angular distribution of particle projections used in the final 3D reconstruction of the EFC. (F) Angular distribution of particle projections used in the final 3D reconstruction of the EFC+F9. Heatmaps indicate particle counts per viewing angle, with red regions representing higher particle densities. (G) Local resolution analysis of the cryo-EM map (contoured at 2.5σ) for the EFC. (H) Local resolution analysis of the cryo-EM map (contoured at 2.5σ) for the EFC+F9. (I) Local resolution analysis of the cryo-EM map (contoured at 6σ) for the EFC. (J) Local resolution analysis of the cryo-EM map (contoured at 6σ) for the EFC+F9. Maps are color-coded according to their respective local resolution values.

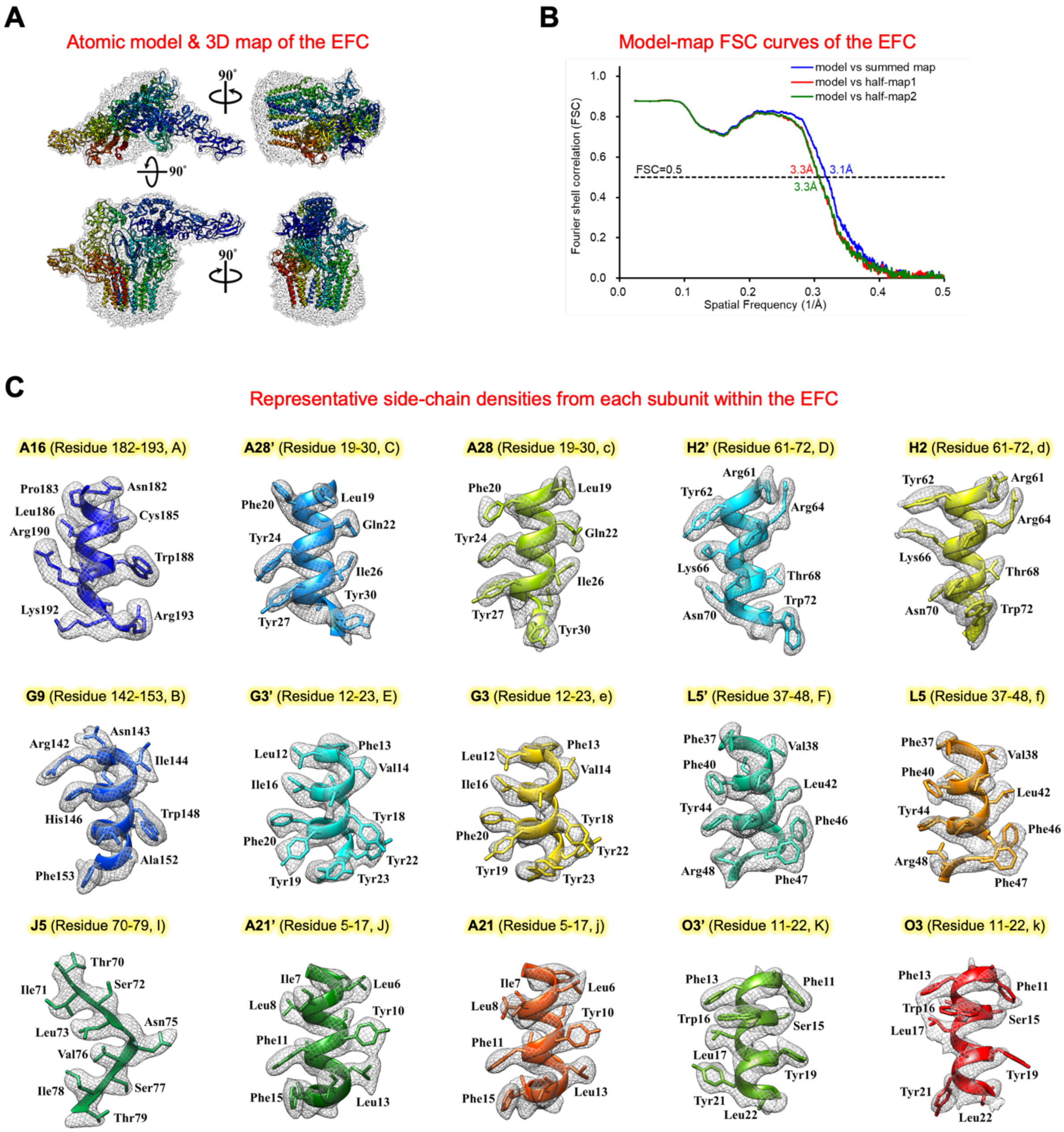
A
Atomic model & 3D map of the EFC
90˚
90˚
90˚
B
Model-map FSC curves of the EFC
model vs summed map
model vs half-map1
model vs half-map2
FSC=0.5
3.3Å
3.1Å
3.3Å
Fourier shell correlation (FSC)
Spatial Frequency (1/Å)
1.0
0.8
0.6
0.4
0.2
0.0
0.0
0.1
0.2
0.3
0.4
0.5
C
Representative side-chain densities from each subunit within the EFC
A16 (Residue 182-193, A)
Pro183 Asn182 Leu186 Cys185 Arg190 Trp188 Lys192 Arg193
A28' (Residue 19-30, C)
Phe20 Leu19 Gln22 Tyr24 Ile26 Tyr30 Tyr27
A28 (Residue 19-30, c)
Phe20 Leu19 Gln22 Tyr24 Ile26 Tyr27 Tyr30
H2' (Residue 61-72, D)
Arg61 Tyr62 Arg64 Lys66 Thr68 Trp72 Asn70
H2 (Residue 61-72, d)
Tyr62 Arg61 Arg64 Lys66 Thr68 Asn70 Trp72
G9 (Residue 142-153, B)
Asn143 Arg142 Ile144 Trp148 His146 Ala152 Phe153
G3' (Residue 12-23, E)
Leu12 Phe13 Val14 Ile16 Tyr18 Phe20 Tyr22 Tyr19 Tyr23
G3 (Residue 12-23, e)
Leu12 Phe13 Val14 Ile16 Tyr18 Phe20 Tyr22 Tyr19 Tyr23
L5' (Residue 37-48, F)
Phe37 Val38 Phe40 Leu42 Tyr44 Phe46 Arg48 Phe47
L5 (Residue 37-48, f)
Phe37 Val38 Phe40 Leu42 Tyr44 Phe46 Arg48 Phe47
J5 (Residue 70-79, I)
Thr70 Ile71 Ser72 Asn75 Leu73 Val76 Ile78 Ser77 Thr79
A21' (Residue 5-17, J)
Ile7 Leu6 Leu8 Tyr10 Phe11 Phe15 Leu13
A21 (Residue 5-17, j)
Ile7 Leu6 Leu8 Tyr10 Phe11 Phe15 Leu13
O3' (Residue 11-22, K)
Phe13 Phe11 Trp16 Ser15 Leu17 Tyr19 Tyr21 Leu22
O3 (Residue 11-22, k)
Phe13 Phe11 Trp16 Ser15 Leu17 Tyr19 Tyr21 Leu22

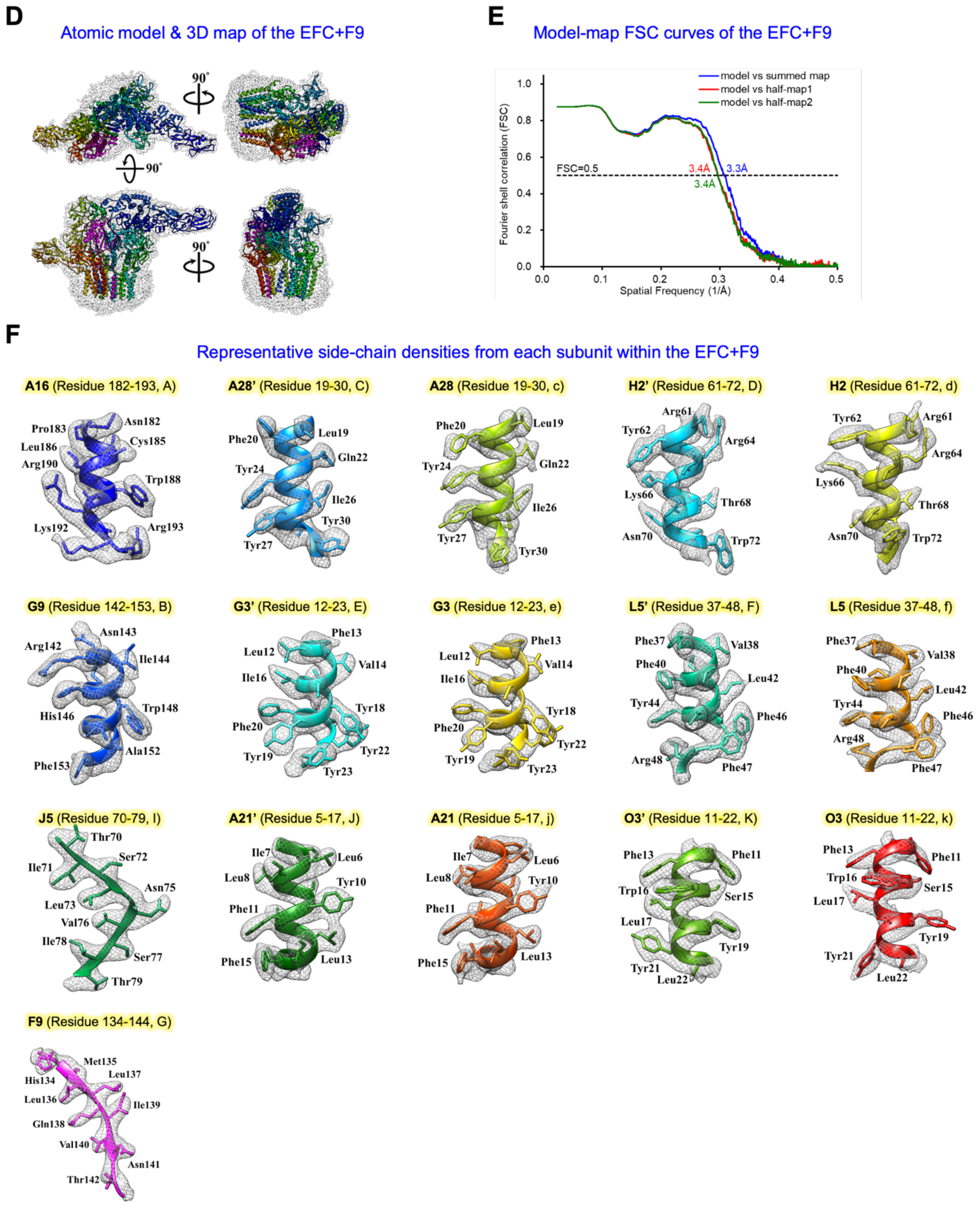

Fig. S5.

Validation of cryo-EM structural models of the EFC complexes. (A) Cryo-EM density map of the EFC, overlaid with the corresponding molecular models. Different subunits are shown in distinct colors, and the cryo-EM density is displayed as a mesh (contoured at 2.5σ). (B) FSC curves (threshold at FSC = 0.5) comparing the molecular model of the EFC against the full reconstructed map (blue) and the two half maps (red and green). (C) Representative side-chain

densities from each subunit within the EFC, demonstrating the quality of the cryo-EM map (gray mesh). Atomic models are shown in different colors for clarity. (D) Cryo-EM density map of the EFC+F9, overlaid with the corresponding molecular models. Different subunits are shown in distinct colors, and the density is displayed as a mesh (contoured at 2.5σ). (E) FSC curves (threshold at FSC = 0.5) comparing the molecular model of the EFC+F9 against the full reconstructed map (blue) and the two half maps (red and green). (F) Representative side-chain densities from each subunit within the EFC+F9, showing the quality of the cryo-EM map (gray mesh). Atomic models are displayed in various colors according to the subunits.

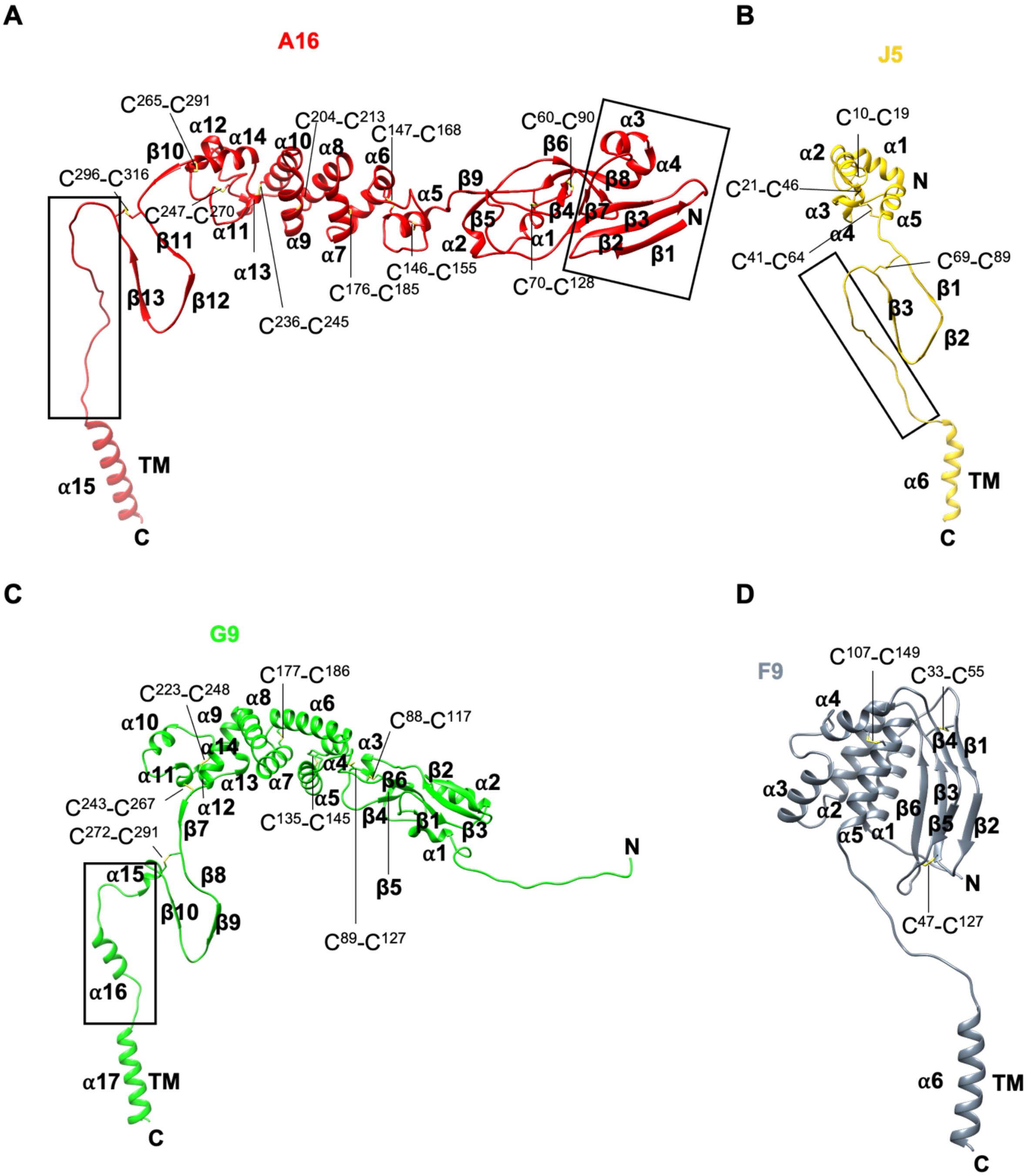
A
A16
C^{265}-C^{291}
C^{204}-C^{213}
C^{147}-C^{168}
C^{60}-C^{90}
C^{296}-C^{316}
C^{247}-C^{270}
C^{146}-C^{155}
C^{176}-C^{185}
C^{70}-C^{128}
C^{236}-C^{245}
α1 α2 α3 α4 α5 α6 α7 α8 α9 α10 α11 α12 α13 α14 α15
β1 β2 β3 β4 β5 β6 β7 β8 β9 β10 β11 β12 β13
N
C
TM
B
J5
C^{10}-C^{19}
C^{21}-C^{46}
C^{41}-C^{64}
C^{69}-C^{89}
α1 α2 α3 α4 α5 α6
β1 β2 β3
N
C
TM
C
G9
C^{177}-C^{186}
C^{223}-C^{248}
C^{88}-C^{117}
C^{243}-C^{267}
C^{135}-C^{145}
C^{272}-C^{291}
C^{89}-C^{127}
α1 α2 α3 α4 α5 α6 α7 α8 α9 α10 α11 α12 α13 α14 α15 α16 α17
β1 β2 β3 β4 β5 β6 β7 β8 β9 β10
N
C
TM
D
F9
C^{107}-C^{149}
C^{33}-C^{55}
C^{47}-C^{127}
α1 α2 α3 α4 α5 α6
β1 β2 β3 β4 β5 β6
N
C
TM

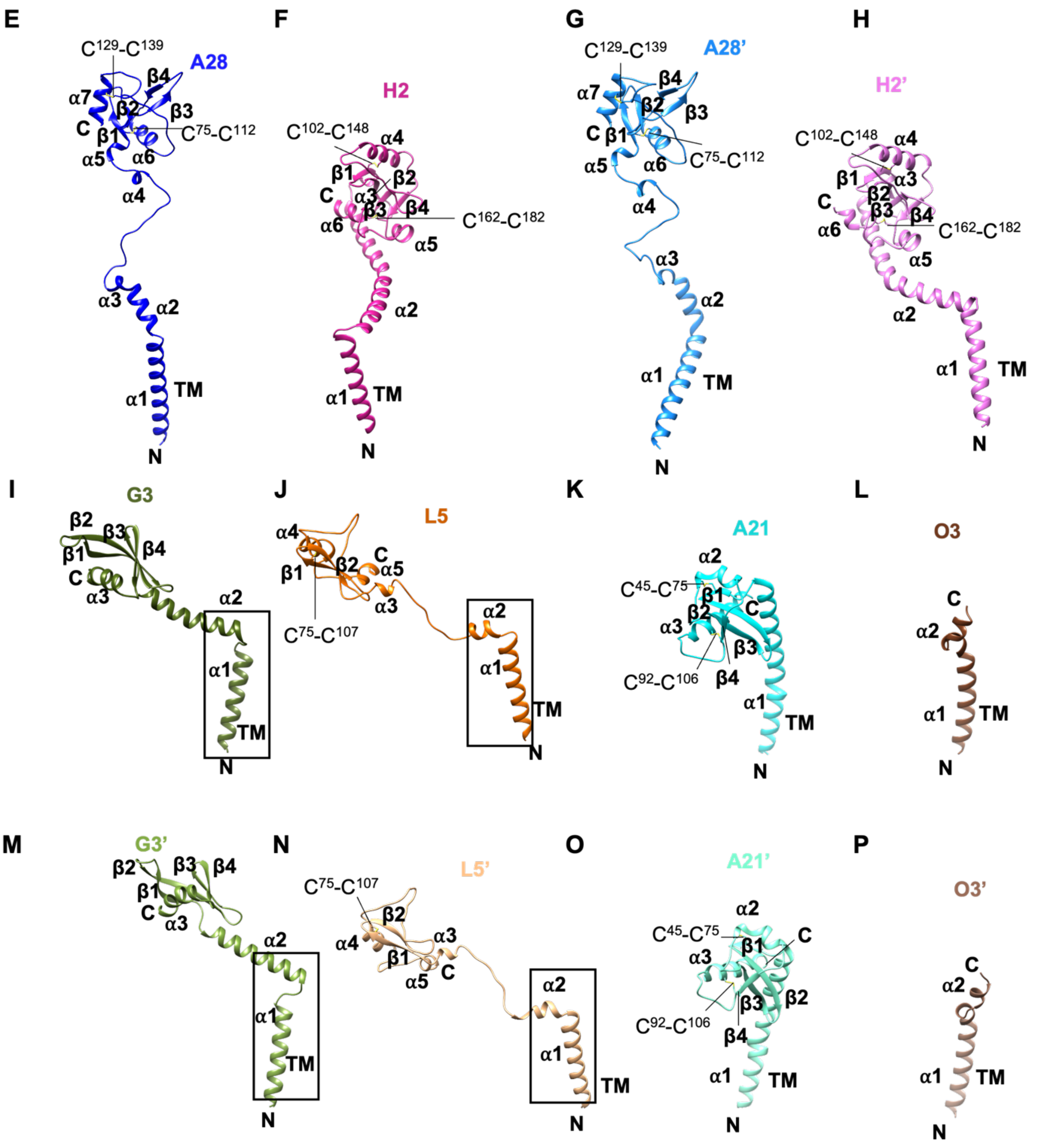

Fig. S6.

Cryo-EM structure of each EFC component. (A-P) Cartoon representation of 16 EFC components. The secondary structure elements α-helices and β-strands are marked. The disulfide bonds are shown in yellow. The N-terminus (N), C-terminus (C), and TM domains are labeled. EFC components are color-coded as in Figure 1C. Regions requiring manual rebuilding due to poor density or conformational variation, including the disordered C-terminal loops of A16, G9, and J5; the A16 N-terminal domain (residues 8-41 and 109-121); and the transmembrane helices of both G3/G3’ and L5/L5’ copies, are boxed.

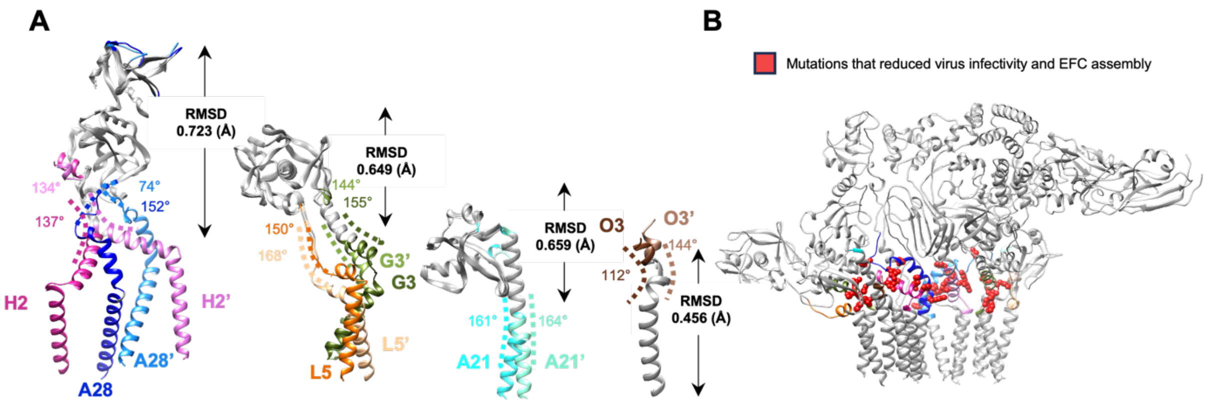

Fig. S7.

Structural alignment of identical EFC subcomplexes and mapping residues that cause loss of infectivity from mutation in the hinge regions. (A) Cartoon representation of structural superposition between A28/H2 and A28'/H2', G3/L5 and G3'/L5', A21 and A21', O3 and O3'. The aligned region of the compared proteins is colored in gray with the RMSD value indicated, and unaligned parts are in colors. (B) Cartoon representation of the EFC with the hinge regions highlighted in colors. Residues that cause loss of virus infectivity when mutated are shown as spheres and color-coded in red.

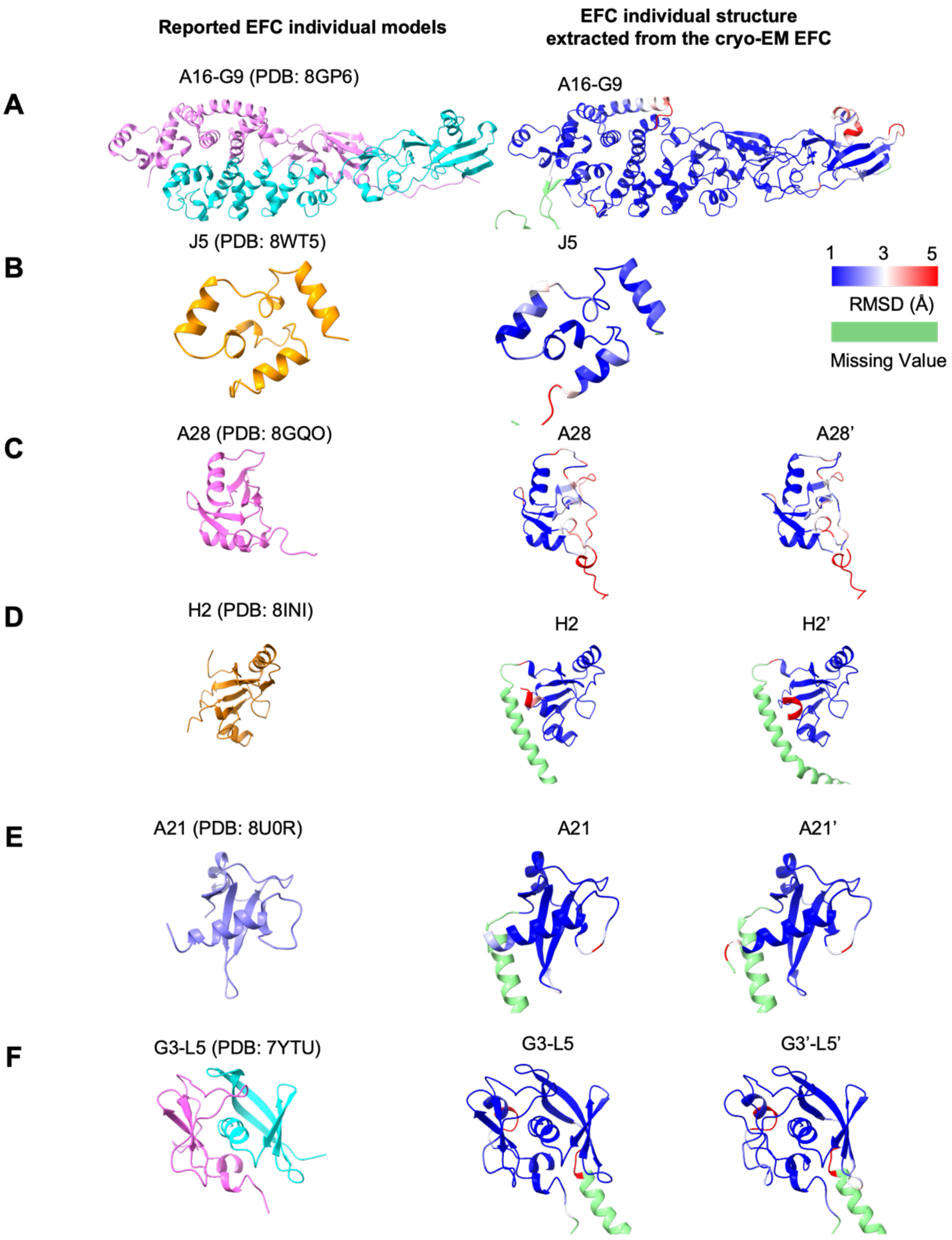

Fig. S8.

Structural comparison of individual EFC components (PDB 9UZO) with previously reported structures. (A-F) EFC components are colored according to the RMSD value (Å) from the comparison pair. Missing values indicate that no corresponding region is present in the previously solved structures.

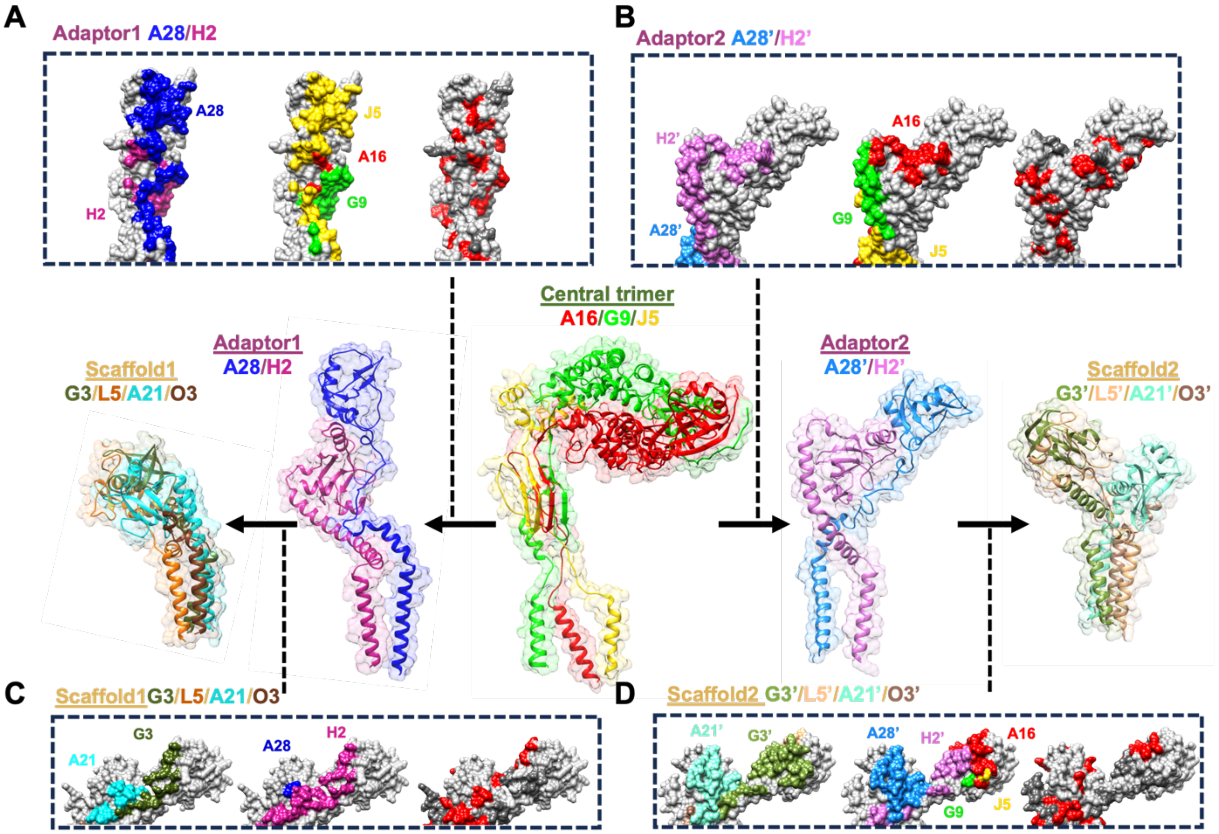

Fig. S9.

Adaptors A28/H2 & A28'/H2' and scaffolds G3/L5/A21/O3 and G3'/L5'/A21'/O3' utilize distinct interfaces to assemble the asymmetrical A16/G9/J5 central trimer. (A-D) Surface representation of the ectodomain of adaptor1 A28/H2 (A), adaptor2 A28'/H2' (B), scaffold1 G3/L5/A21/O3 (C), and scaffold2 G3'/L5'/A21'/O3' (D). The surface is color-coded based on contact residue (left panel), the binding counterparts of contact residues (middle panel), and mutants that lose virus infectivity (right panel). Residues that cause loss of virus infectivity when mutated are color-coded red, and the EFC proteins are color-coded as in Figure 1C.

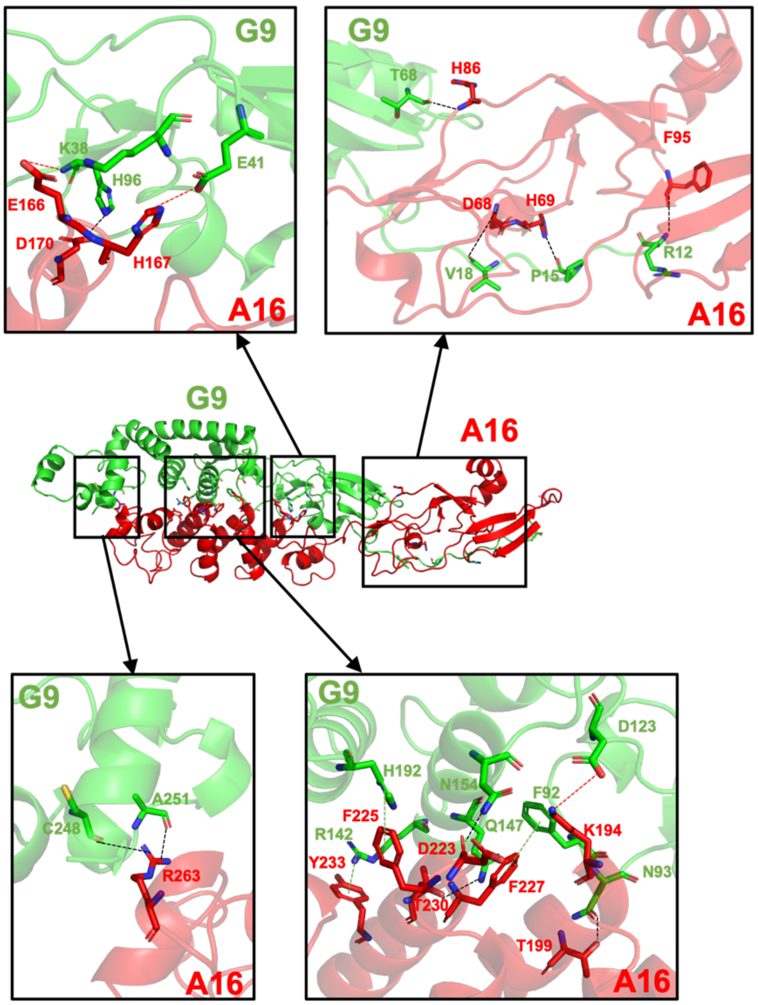

Fig. S10.

Contact interface of G9 and A16 in the ectodomain. The contact interface between A16 and G9 in the ectodomain is highlighted and magnified. Residues that participate in the interaction are displayed in stick form and labeled. The bonds between paired residues are represented by dashed lines, with black indicating hydrogen bonds, red indicating ionic interactions, and green indicating π-π and π-cation interactions.

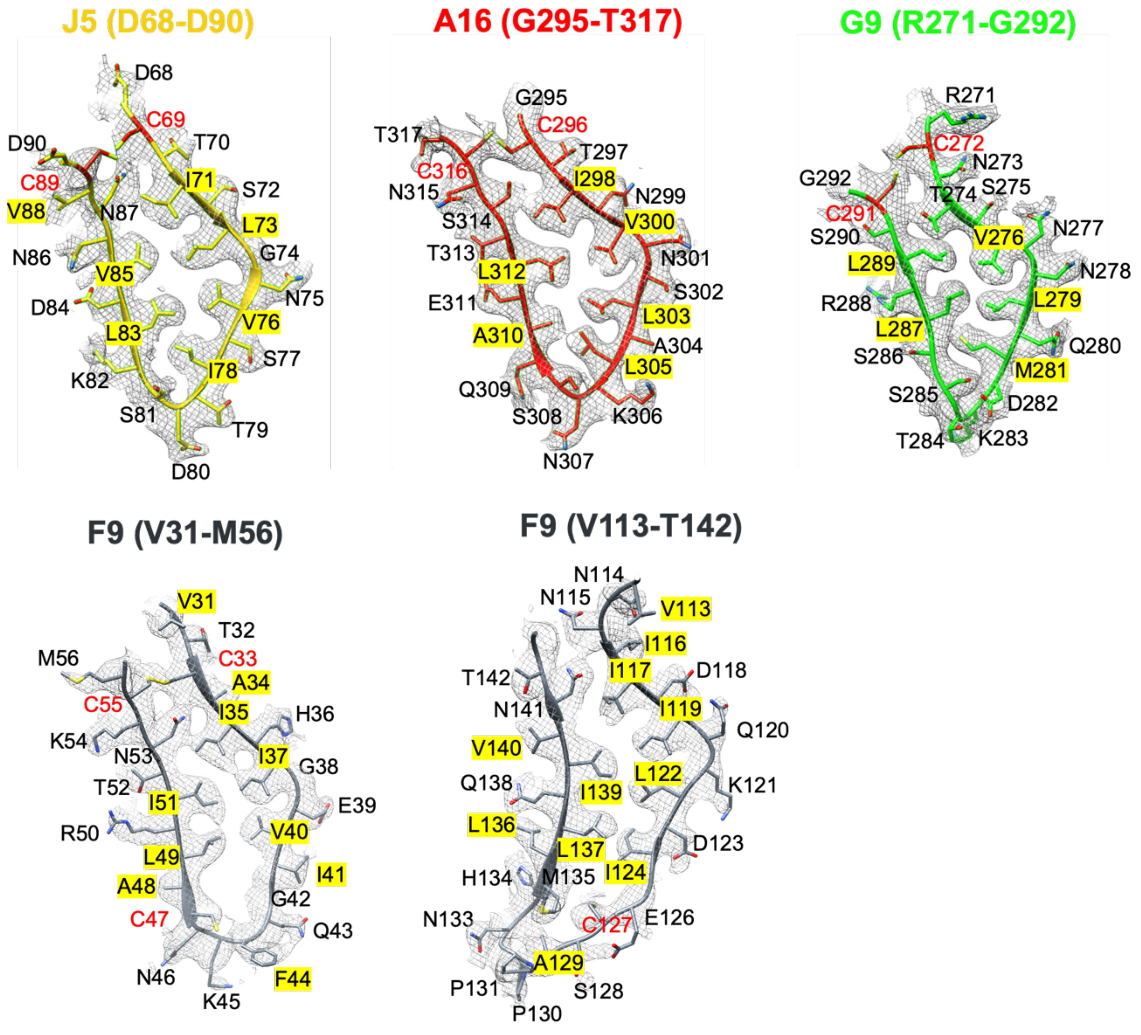

Fig. S11.

Residues forming the Delta motif in A16, G9, J5, and F9. Close-up view of the hydrophobic residues in the Delta motifs of A16, G9, J5, and F9. Motifs are shown as ribbon diagrams overlaid with the corresponding cryo-EM density map (contoured at 4.5σ). Nitrogen, oxygen, and sulfur atoms are colored in blue, red, and yellow, respectively.

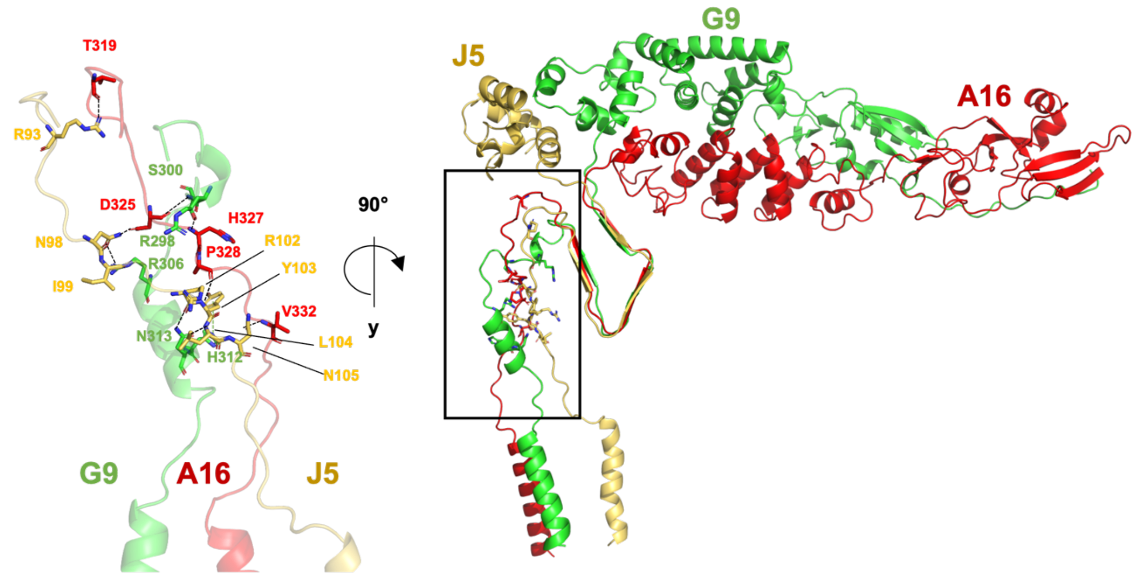

Fig. S12.

Zoomed in view of the hair-braiding interaction between A16, G9, and J5. The contact interface between A16, G9, and J5 in the lower region of the heterotrimers is boxed and magnified. Residues that participate in the interaction are displayed in stick form and labeled. The hydrogen bonds between paired residues are represented by dashed lines.

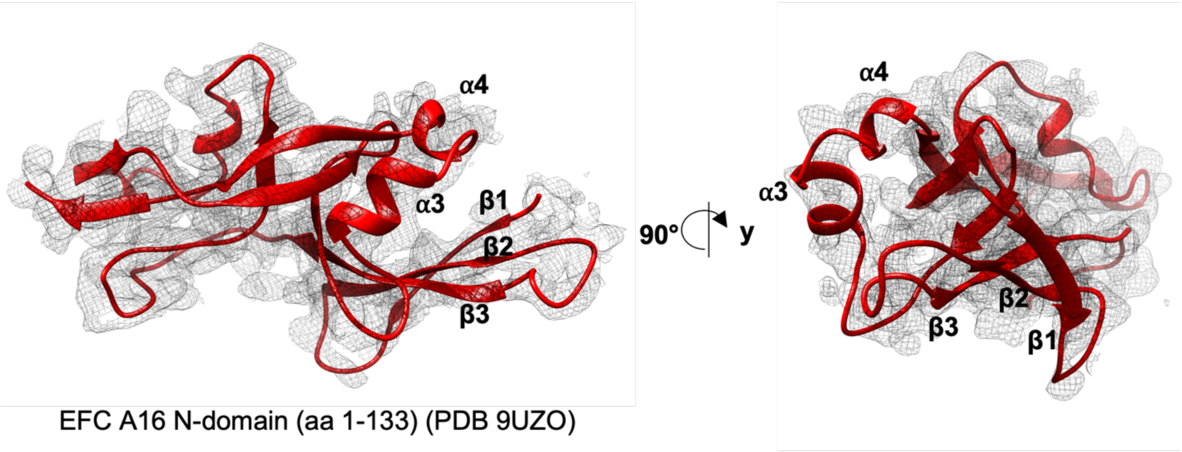

Fig. S13.
The N-domain of A16 (aa 1-133) in the EFC (PDB 9UZO). Cartoon representation of A16 N-domain viewed from two directions overlaid with the corresponding cryo-EM density map (contoured at 2.5σ). The secondary structure elements are labeled.

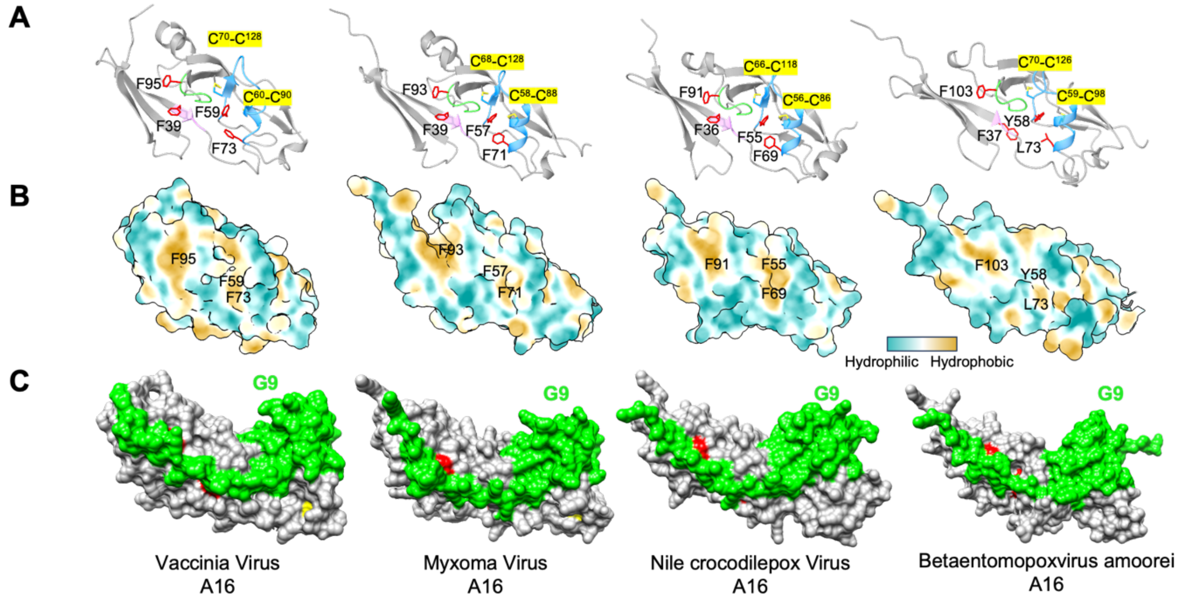

Fig. S14.

Bioinformatic analysis of A16 identified a fusion domain candidate. (A) Cartoon representation of N-terminal domains of VACV A16 and A16 orthologs in indicated poxviruses. The three conserved motifs in Figure 5B are color-coded as follows: motif 1 (lavender), motif 2 (blue), and motif 3 (green). The conserved aromatic residues are shown in red sticks. The A16 orthologs in poxviruses are AlphaFold predicted models. (B) Surface hydrophobicity of N-terminal domains shown in (A) with conserved aromatic residue labeled. (C) Surface presentation of N-terminal domains of A16-G9 dimer complex of VACV and indicated poxviruses with A16 in gray, G9 in green, and the conserved aromatic residue in red. The A16-G9 dimer complex of poxviruses orthologs are predicted by AlphaFold.

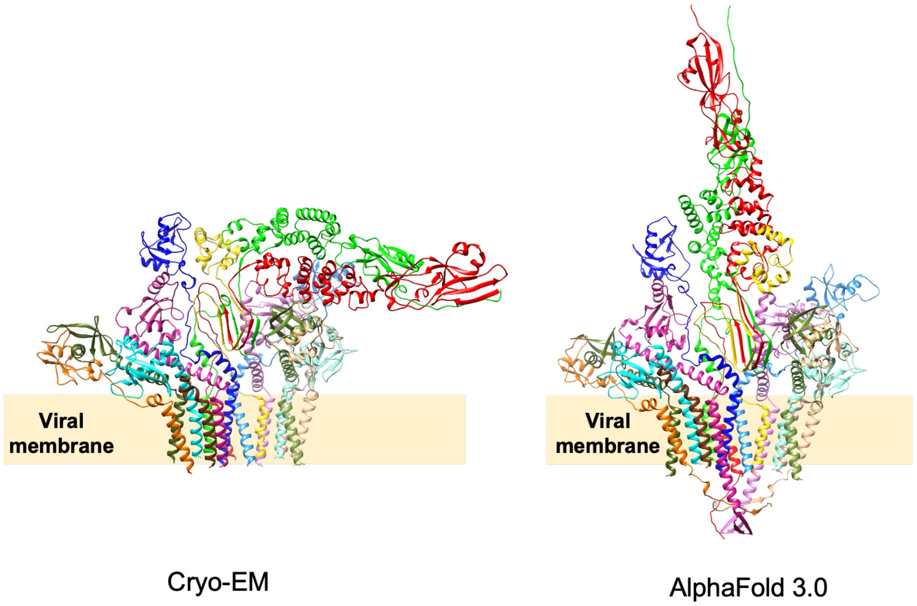

Fig. S15.
Structural differences between the cryo-EM structure of the EFC and the AlphaFold 3.0 model. Cryo-EM structure of the EFC (left) and the AlphaFold 3.0 predicted model (right). The N-terminal ectodomains of A16/G9 in the cryo-EM EFC lie in parallel with the viral membrane, whereas the equivalent positions in A16/G9 in the AlphaFold model are perpendicular to the viral membrane. EFC proteins are color-coded as in Figure 1C.

Table S1.
Cryo-EM data collection, refinement, and validation statistics.

	**EFC (EMD-64647) (PDB-9UZO)**	**EFC+F9 (EMD-64648) (PDB-9UZP)**
**Data collection**		
EM equipment	Titan Krios	Titan Krios
Voltage (kV)	300	300
Cs (mm)	2.7	2.7
Magnification (nominal)	81,000	81,000
Detector	K3	K3
Pixel size (Å)	0.5305	0.5305
Electron exposure (e^{-}/Å^2)	~ 50.4	~ 50.4
Exposure time (s)	1.34	1.34
Frames (no.)	50	50
Defocus range (µm)	-0.13 ~ -4.99	-0.13 ~ -4.99
**Reconstruction**		
Software	cryoSPARC	cryoSPARC
Micrographs stacks (no.)	17,298	17,298
Final particle images (no.)	526,492	474,787
Symmetry imposed	C1	C1
Map final resolution (Å)†	2.98	3.05
Map sharpening B-factor (Å2)	-82.5	-80.8
**Atomic modeling**		
Software	Coot & Phenix	Coot & Phenix
Number of protein residues	2,171	2,365
Number of ligands	0	0
Number of atoms	17,552	19,067
Map CC (around atoms)*	0.84	0.81
RMSD bond lengths (Å)	0.006	0.004
RMSD bond angles (°)	0.975	0.931
Clash score*	5.85	5.62
Ramachandran favored (%)*	96.92	97.64
Ramachandran allowed (%)*	3.08	2.36
Ramachandran outliers (%)*	0	0
Rotamer outliers (%)*	0	0
C_β deviations*	0	0
MolProbity score*	1.50	1.38

†According to FSC=0.143
* According to the criteria of Chen *et al.*, 2010 (https://doi.org/10.1107/s0907444909042073) (*83*)

Table S2.

Infectivity of mutant VACV containing mutations in G9, J5, A28, and H2 ORFs.

	Mutation	Infectivity				Reference
		75-100%	50-75%	25-50%	<25%	
G9	ΔG2-P19				V	(*49*)
	R12A	V				
	D21A	V				
	E22R				V	
	ΔN26-L39				V	
	D28K29H31D32A4		V			
	H31A	V				
	K38A	V				
	H44Y	V				
	G46A				V	
	D51K52E53D54K55K57K58R59A8	V				
	Y69A			V		
	E62A	V				
	R78A				V	
	H96Y97W98A3				V	
	H96A		V			
	Y97A	V				
	W98A			V		
	K99K100D101G102K103A5	V				
	K107E109E110A3	V				
	P119A	V				
	D120H122D123K126D128A5				V	
	D120A	V				
	H122A		V			
	D123A		V			
	K126A	V				
	D128A	V				
	R142A	V				
	H146A	V				
	W148G		V			
	H191H192R194A3		V			
	E199D200K202E203D206A5		V			
	D215K219R222A3		V			
	E233E239A2	V				
	P240R241E242W244A4				V	
	P240A	V				
	R241A	V				
	E242A			V		
	W244A	V				
	Δ273-S290::A3				V	
	L279A	V				
	K308H312K315H316A4	V				
	H312A	V				
	H316A	V				
	F318A	V				
J5	D3A	V				(*40*)
	D11K14A2	V				
	D3D11K14A3	V				

	SW* (11-18)	V				
	SW* (22-40)		V			
	P38Y39Y40A3		V			
	W42Y43A2		V			
	P38Y39Y40W42Y43A5			V		
	SW* (42-63)	V				
	S56I	V				
	N65A	V				
	SW* (70-88)		V			
	N87A	V				
	SW* (90-110)			V		
	Y103Q107A2	V				
A28	W73A		V			(*39*)
	R74A	V				
	F89A		V			
	G90A				V	
	D56A		V			
	D56R58A		V			
	D56R58D114D119A			V		
	V62A	V				
	D61V62N63D64A			V		
	K72A			V		
	D68K72R74A				V	
	F91A	V				
	N99T100R101S102A	V				
	G96N99T100R101S102	V				
	N122A	V				
	Y126N127A		V			
	N122Y126N127A			V		
	Q22A	V				
	Y27Y30A				V	
	I33A				V	
	K34E35A	V				
	H40E44A				V	
	K47A		V			
H2	D95D96E99K101A	V				(*35*)
	F105A	V				
	D104F105G106A		V			
	K116K117A	V				
	L120P121A		V			
	R125A	V				
	R126A		V			
	R125R126A				V	
	G127A	V				
	G129A		V			
	G127G129A			V		
	D130A			V		(*31*)
	W132A			V		
	W132E			V		
	K135A	V				
	K136A		V			
	K139A	V				
	D141A				V	

	Q146A		V			
	K153H154K155A	V				
	H154R	V				
	H180A	V				
	H154H180R	V				
	G163A	V				
	E169A	V				
	G171G174A				V	
	Y172A			V		
	W181A		V			
	C162A		V			
	L170E			V		(*35*)
	G171A		V			
	G171E				V	
	Y172A			V		
	S173A	V				
	G174A	V				
	G174E			V		
	G171G174A			V		
	Y175A		V			
	F176E	V				
	C182A			V		
	C162C182A	V				
	S42A		V			
	E58A		V			
	R64A	V				
	I65A			V		
	K66A		V			
	R64I65K66A				V	
	W72A		V			
	I65W72A				V	
	K79A		V			
	E83S84D85R86G87R88A			V		
	D95D96E99K101A	V				
	F105A	V				
	D104F105G106A		V			
	K116K117A	V				
	L120P121A		V			
	R125A	V				

*SW denotes substitution of indicated region with the corresponding sequence from ***Betaentomopoxvirus amoorei***.

Table S3.
A16 orthologs used for multiple sequence alignment in this study.

**Abbr.**	**Virus**	**accession**	**Protein**
MsEPV	*Melanoplus sanguinipes entomopoxvirus*	NP_048161.1	A16 (OPG143)
AmEPV	*Betaentomopoxvirus amoorei*	NP_064900.1	A16 (OPG143)
CRV	*Nile crocodilepox virus*	YP_784329.1	A16 (OPG143)
ORFV	*Orf virus*	ASY92389.1	A16 (OPG143)
SPV	*Seal parapoxvirus*	YP_009389379.1	A16 (OPG143)
SOPV	*Sea otter poxvirus*	YP_009480644.1	A16 (OPG143)
PPV	*Pigeonpox*	YP_009046412.1	A16 (OPG143)
MMPV	*Murmansk poxvirus*	YP_009408314.1	A16 (OPG143)
RCNV	*Raccoonpox*	YP_009143444.1	A16 (OPG143)
EfPV	*Eptesipox virus*	YP_009408064.1	A16 (OPG143)
COTV	*Cotia virus*	YP_005296313.1	A16 (OPG143)
MYXV	*Myxoma virus*	AGU99789.1	A16 (OPG143)
SWPV	*Swinepox virus*	QQG31595.1	A16 (OPG143)

Movie S1. (separate file)
Cryo-EM structures of the multiprotein machinery VACV EFC and VACV EFC+F9.

Data S1. (separate file)
Accession numbers for A16, G9, and J5 orthologs in different members of the *Poxviridae* family.
